# Deceleration of Fusion–Fission Cycles Improves Mitochondrial Quality Control during Aging

**DOI:** 10.1371/journal.pcbi.1002576

**Published:** 2012-06-28

**Authors:** Marc Thilo Figge, Andreas S. Reichert, Michael Meyer-Hermann, Heinz D. Osiewacz

**Affiliations:** 1 Applied Systems Biology, Leibniz-Institute for Natural Product Research and Infection Biology – Hans-Knöll-Institute and Friedrich Schiller University, Jena, Germany; 2 Frankfurt Institute for Advanced Studies, Frankfurt am Main, Germany; 3 Department of Systems Immunology, Helmholtz Centre for Infection Research, Braunschweig, Germany; 4 Mitochondrial Biology, Medical School, Goethe University Frankfurt am Main, Frankfurt am Main, Germany; 5 Mitochondrial Biology, Buchmann Institute for Molecular Life Sciences, Frankfurt am Main, Germany; 6 Faculty for Biosciences, Molecular Developmental Biology, Cluster of Excellence Macromolecular Complexes, Goethe University, Frankfurt am Main, Germany; University of Auckland, New Zealand

## Abstract

Mitochondrial dynamics and mitophagy play a key role in ensuring mitochondrial quality control. Impairment thereof was proposed to be causative to neurodegenerative diseases, diabetes, and cancer. Accumulation of mitochondrial dysfunction was further linked to aging. Here we applied a probabilistic modeling approach integrating our current knowledge on mitochondrial biology allowing us to simulate mitochondrial function and quality control during aging *in silico*. We demonstrate that cycles of fusion and fission and mitophagy indeed are essential for ensuring a high average quality of mitochondria, even under conditions in which random molecular damage is present. Prompted by earlier observations that mitochondrial fission itself can cause a partial drop in mitochondrial membrane potential, we tested the consequences of mitochondrial dynamics being harmful on its own. Next to directly impairing mitochondrial function, pre-existing molecular damage may be propagated and enhanced across the mitochondrial population by content mixing. In this situation, such an infection-like phenomenon impairs mitochondrial quality control progressively. However, when imposing an age-dependent deceleration of cycles of fusion and fission, we observe a delay in the loss of average quality of mitochondria. This provides a rational why fusion and fission rates are reduced during aging and why loss of a mitochondrial fission factor can extend life span in fungi. We propose the ‘mitochondrial infectious damage adaptation’ (MIDA) model according to which a deceleration of fusion–fission cycles reflects a systemic adaptation increasing life span.

## Author Summary

Mitochondria are organelles that play a central role as ‘cellular power plants’. The cellular organization of these organelles involves a dynamic spatial network where mitochondria constantly undergo fusion and fission associated with the mixing of their molecular content. Together with the processes of mitophagy and biogenesis of mitochondrial mass, this results into a cellular surveillance system for maintaining their bioenergetic quality. The accumulation of molecular damage in mitochondria is associated with various human disorders and with aging. However, how these processes affect aging and how they can be reconciled with existing aging theories is just at the beginning to be considered. Mathematical modeling allows simulating the dynamics of mitochondrial quality control during aging *in silico* and leads to the ‘mitochondrial infectious damage adaptation’ (MIDA) model of aging. It reconciles a number of counterintuitive observations obtained during the last decade including infection-like processes of molecular damage spread, the reduction of fusion and fission rates during cellular aging, and observed life span extension for reduced mitochondrial fission. Interestingly, the MIDA model suggests that a reduction in mitochondrial dynamics rather than being merely a sign or even cause of aging, may actually reflect a systemic adaptation to prolong organismic life span.

## Introduction

Mitochondria are double-membrane enclosed organelles that fulfill a number of essential cellular roles including oxidative phosphorylation, thermogenesis, iron-sulfur cluster biogenesis, biosynthesis of heme, certain lipids and amino acids, and regulation of apoptosis. They are semiautonomous organelles which are depending on the expression of both, nuclear as well as mitochondrially encoded genes. For example, in humans the mitochondrial DNA (mtDNA) which exists in several 100 s to 1000 s copies per cell encodes two rRNAs, a set of tRNAs and 13 subunits of the electron transport chain (ETC). About 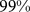 of all mitochondrial proteins are encoded by the nucleus, synthesized in the cytoplasm and imported into mitochondria.

Mitochondrial dysfunction is linked to a number of human disorders including neurodegenerative diseases, myopathies, obesity, diabetes, and cancer (for review see [1], [2]). Moreover, several theories aiming to explain aging in eukaryotes ascribe a crucial role to mitochondria. One hypothesis dominating the last decades, known as the ‘mitochondrial free radical theory’ (MFRT) of aging proposed by Harman [3] states that reactive oxygen species (ROS), predominantly generated within the mitochondrial ETC, cause molecular damage in a cumulative manner. Later refinements of this theory suggest a vicious cycle to occur since mtDNA encoding essential subunits of the ETC are damaged by ROS [4]. Although the aforementioned ‘refined’ MFRT is currently hotly debated and specifically the existence of a vicious cycle is unclear [5]–[9], it is undisputed that oxidative stress contributes to and mitochondrial dysfunction is involved in aging processes (for reviews see [6], [10]–[12]). However, the vast majority of studies undertaken so far have addressed mainly how oxidative stress is generated within mitochondria, what are the cellular and molecular targets being damaged, and how these processes contribute to aging. A different point-of-view has gained increasing attention recently, namely, what are the molecular mechanisms that reduce the amount of mitochondrial dysfunction and ROS formation. Thus, rather than focusing on the formation of molecular damage our main interest has now shifted to the mechanisms ensuring its removal which appears equally important to the aging process. Mitochondrial quality control has recently been linked by several studies to the astonishing dynamic organization of the mitochondrial network [13], [14] and to the selective removal of dysfunctional mitochondria by mitophagy [15]–[21].

Mitochondria constantly undergo fusion and fission events [13], [14]. Impairment of the dynamic behavior is linked to a range of neurodegenerative diseases and aging [22]–[34]. Mitochondrial fusion was proposed as a mechanism primarily mediating content mixing and by that allowing inter-mitochondrial complementation compensating for missing or dysfunctional gene products of individual mitochondria [35], [36]. Impairment of mitochondrial function was shown to strongly inhibit mitochondrial fusion both in yeast as well as in mammalian cells [16], [17], [37]. Consequently, under bioenergetically compromised conditions, the tubular network of mitochondria transforms into individual, spatially separated, mitochondria exhibiting a rather fragmented appearance. The selective loss of the fusion capacity of damaged mitochondria, but not of functional ones, could act as a mechanism to distinguish non-functional from functional mitochondria on a morphological basis [16]–[18]. This in turn might prevent or minimize further molecular damage as, first, dysfunctional mitochondria are spatially separated from the intact mitochondrial network, and, second, the smaller size itself might be prerequisite for their selective removal by autophagy a process known as mitophagy. Indeed, impairing mitochondrial fission in mammalian cells inhibited mitophagy and, conversely, inhibiting autophagy increased the levels of depolarized mitochondria and led to reduced maximal respiration [19]. Taken together, these measurements indicate that fusion occurs selectively between functional mitochondria and that fission acts as to continuously separate mitochondria from each other facilitating the isolation and the subsequent removal of damaged, non-fusogenic mitochondria. The mitochondrial life cycle thus appears to represent an efficient mechanism ensuring the molecular quality of the cellular ensemble of mitochondria [38]. A possible link of such a quality control system to aging was also discussed in more detail recently [39].

Although the general view that mitochondrial dynamics and mitophagy are beneficial for maintaining mitochondrial integrity is supported by numerous studies, it should be noted that basically all studies in mammalian cells rely on tumor cell lines and/or on conditions in which mitochondrial functions are affected by non-physiological conditions (*e.g.*, overexpression of PARKIN and the addition of the uncoupler CCCP). It is by far not clear whether all conclusions derived from such systems can indeed be transferred to primary tissues or entire organisms. This concern is further strengthened by findings demonstrating that mitochondrial dynamics is tightly linked to the cell cycle as e.g. hyperfusion of mitochondria was found to be required for progression from G1 to S phase and mitochondrial fission is promoted during mitosis [40]–[42]. Unfortunately, the entire field is lacking quantitative data on the rates of mitochondrial fusion and fission as well as of mitophagy in primary tissues under normal and pathological conditions. Moreover, there are a number of observations that clearly appear counterintuitive considering such a mitochondrial quality control system. For example, the pathogenesis of certain mitochondrial diseases caused by mtDNA mutations (*e.g.*, MELAS, mitochondrial encephalomyopathy, lactic acidosis, and stroke-like episodes syndrome; MERRF, myoclonic epilepsy with ragged red fibers syndrome) is not well understood. These diseases develop progressively and the severity of the symptoms is positively correlated to age and degree of heteroplasmatic prevalence of mutated mtDNA molecules (for review see [1]). However, the mutational load appears to be highly variable between different cells, tissues, or even between mother and affected offspring. Clonal expansion of various mtDNA haplotypes in higher eukaryotes is frequently observed [12], [43]–[46]. Another counterintuitive observation is that in a cellular model of aging the rate of both fusion and fission events become reduced by more than 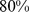 in aged cells compared to young cells [47]. As aging is well known to be associated with the accumulation of molecular damage, an increase in mitochondrial dynamics would be expected to better cope with this situation. Moreover, it was shown that ablation of mitochondrial fission extends the life span of the two fungal species, *Podospora anserina* and *Saccharomyces cerevisiae* [34]. These experiments reveal that a reduced fission rate retards aging without impairing fitness and fertility of the mutants which also appears incompatible with a fission-dependent mitochondrial quality control mechanism. Taken together, there are a number of apparent discrepancies suggesting that mitochondrial quality control and its age-dependent regulation might be more complex than initially assumed.

This prompted us to apply a systems biology approach which aims to integrate and to challenge current views in mitochondrial biology during aging into a probabilistic model. This model considers alterations of mitochondrial dynamics as an adaption to the accumulation of molecular damage in aging cells and thus differs from the Monte Carlo approach [48] or the ‘organelle control’ theory [49] reported previously. We investigated the hypothesis that mitochondrial dynamics itself could be harmful, for example in situations when molecular damage has already accumulated to some degree and further content mixing would lead to an infection-like phenomenon causing molecular damage to spread across the entire mitochondrial population. Thus, the fission processes itself could, in addition to its beneficial effect on mitochondrial quality control under certain conditions impose molecular damage to mitochondria. This is supported by the observation that mitochondrial membrane potential was reduced in a significant fraction of mitochondria subsequent to a fission event in mammalian cells [19]. Our simulations suggest that a reduction in mitochondrial dynamics rather than being merely a sign or even putative cause of aging, may actually reflect a systemic adaptation to slow down the molecular damage and its propagation by cycles of fusion and fission to prolong cellular function and organismic life span. Here we present and discuss this concept as the ‘mitochondrial infectious damage adaptation’ (MIDA) model.

## Models

**Figure 1:**
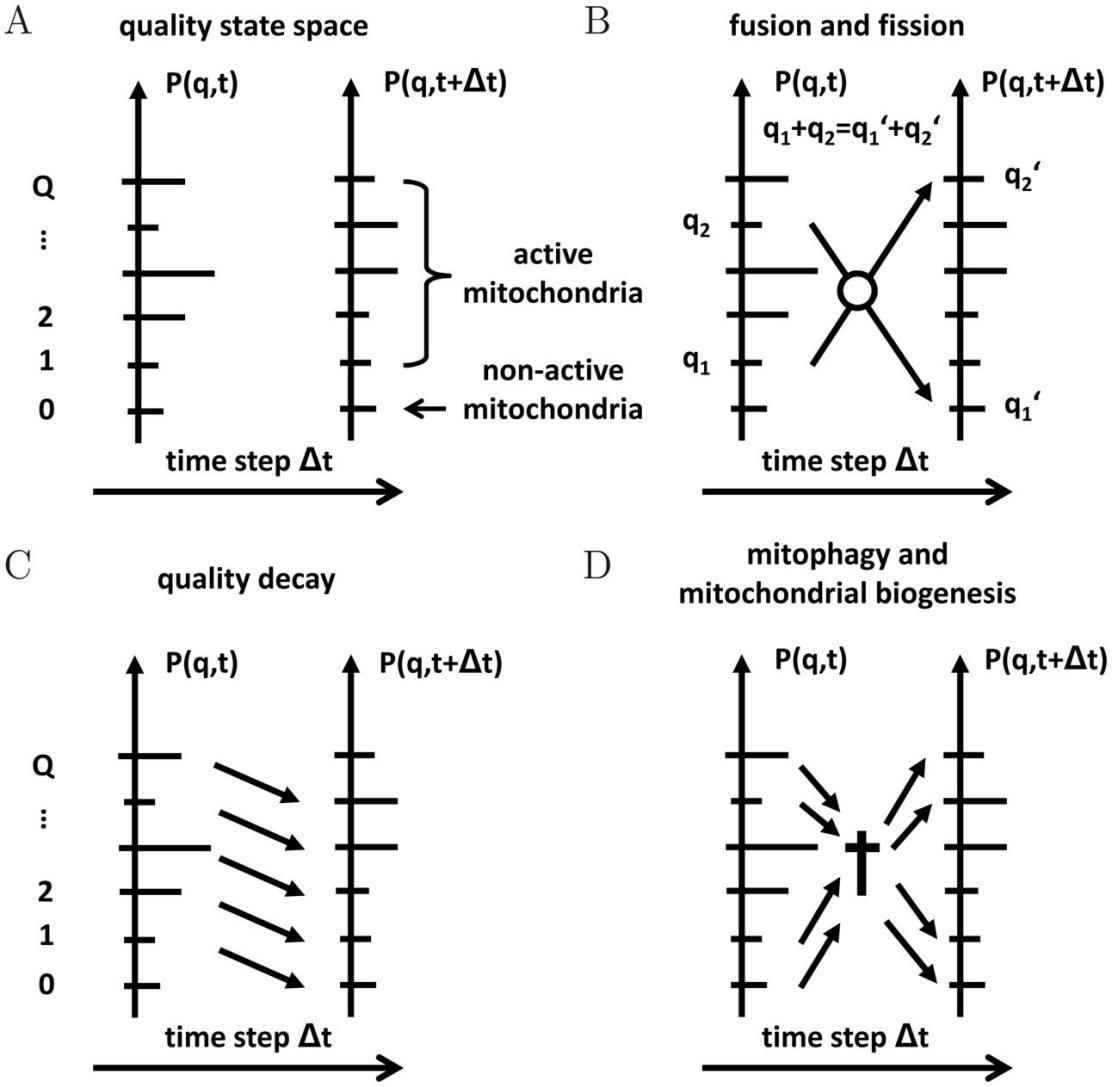
Schematic representation of the quality state-space and processes in the reference simulation. *(A)* The quality state-space consists of a finite number of discrete states 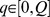 referring to the functional quality of mitochondria with distribution 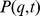. *(B)* Mitochondria with functional quality 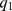 and 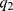 undergo fusion–fission events into quality levels 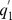 and 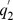 under functional quality conservation: 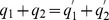. *(C)* Mitochondria are subjected to molecular quality decay resulting in an overal downward-shift of 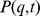. *(D)* Mitophagy gives rise to a loss of mitochondria in quality state 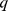, while biogenesis of mitochondrial mass results in a gain of mitochondria in quality state 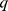. Imposing homeostatic conditions, the norm of 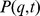 remains constant at all times.

The dynamics of the cellular ensemble of mitochondria within a quality state-space is described by a probabilistic modeling approach. A schematic representation of this space is given in Fig. 1A. Mitochondria are distributed on a ladder of levels representing their functional quality by the discrete number 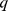. Since the functional quality of mitochondria is limited, we consider the quality state-space to be finite with 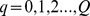, where 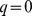 and 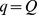, respectively, denote the state of lowest and highest quality. In the course of time, mitochondria are subjected to various processes, *e.g.* fusion and fission, mitophagy, and biogenesis of mitochondrial mass as depicted in Fig. 1B–D. These processes give rise to the time evolution of the mitochondrial distribution in the quality state-space. We calculate the time evolution for the probability 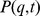 to find mitochondria in a particular quality state using the master equation approach [50].

### Master equation approach in quality state space

We derive the master equation for the time evolution of the probability distribtuion 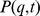 by considering all possible processes that contribute to the probability of finding mitochondria in quality state 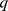 at time 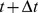:
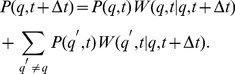
(1)The first term on the right-hand side accounts for the case where the system is in quality state 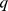 at time 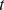 with probability 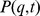 and remains in this state during the time-interval 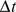. The probability 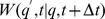 refers to the transition from state 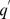 at time 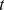 to 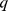 during the time interval 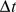. For 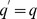, 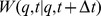 denotes the probability of making no transition during 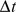. The second term on the right-hand side in Eq. (1) refers to all possible transitions during 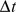 from quality states 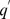 to 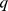 with 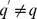.

Representing 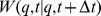 by its counter transition probability, 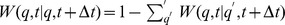, we can rewrite Eq. (1) as:
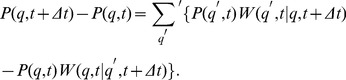
(2)After division of both sides by the time interval 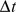, we obtain in the limit 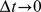 the master equation for the time evolution of 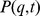 in the generic gain-loss structure:
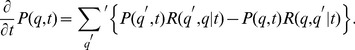
(3)Here, 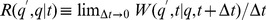 denotes transition rates that are to be determined for each process of the mitochondrial dynamics. In what follows, we determine the contributions for each of these processes separately and then add them up to numerically solve the master equation for 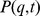. Starting from a normalized probability distribution at 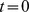, the norm
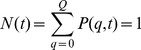
(4)is conserved at all times.

### Fusion–fission events

Mitochondria undergo cycles of fusion and fission by which they exchange their molecular content such that the mitochondrial distribution in quality state-space is altered. As the time between fusion and fission of two mitochondria is typically much shorter than the time that the next fusion event follows a fission event, we consider fusion and fission as paired consecutive events consistent with reported experimental evidence [19]. This approximation is justified for large time scales on which we focus in this study. Furthermore, as shown in Fig. 1B, we impose the condition that the total functional quality of the involved mitochondria is conserved. Thus, two mitochondria in quality state 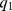 and 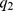 are fusing and subsequently undergo fission into two mitochondria of functional quality 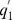 and 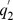, such that 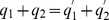.

In practice, we start by computing the list 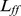 of all possible fusion and fission events, 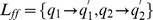, that fulfill the quality conservation condition. In order to avoid double counting of processes, we consider the quality states in the ranges 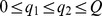 and 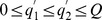. Changes of the mitochondrial distribution in quality state 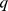 are now easily identified by searching the list 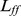. As depicted in Fig. 1B, for 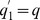 or 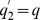, the fusion–fission process ends up with one mitochondrion in quality state 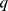 from two fusing mitochondria in quality states 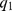 and 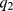. These processes give rise to the gain term in the master equation for fusion–fission events under quality conservation:
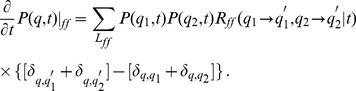
(5)Here, we introduced the fusion–fission rate 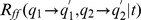 that may depend on time and on the involved quality states. Furthermore, we introduced the Kronecker delta, with 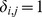 for 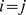 and 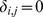 for 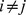, to distinguish the different kinds of transitions. The second term on the right-hand side in Eq. (5) refers to the case where mitochondria of quality state 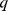, either 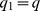 or 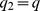, fuse and undergo fission. These are loss processes of quality state 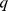 and, therefore, enter the master equation with a minus sign.

### Decay of functional quality

In addition, we account for permanently, yet modestly, ongoing damage to molecular constituents of mitochondria over time. This represents the permanent accumulation of *e.g.* oxidative damage and hydrolytic degradation of proteins, lipids and nucleic acids, but also includes partial misfolding and aggregation of proteins, which overall cause a gradual decline in the amount of active molecules. This process is schematically shown in Fig. 1C and the corresponding master equation is given by:
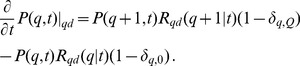
(6)Here, the first term on the right-hand side represents the gain in quality state 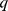 due to mitochondrial quality stepping down the quality ladder from 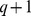. This process requires 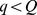, which is taken care of by the Kronecker delta. Similarly, the second term on the right-hand side of Eq. (6) describes the process of mitochondria quality stepping down the quality ladder from 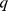 to 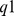, a process which can only occur for 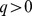. Thus, natural decline of molecular integrity gives rise to a functional quality-shift of the probability distribution 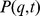 from higher to lower quality states with quality decay rate 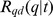.

### Mitophagy and mitochondrial biogenesis

Mitophagy leads to the removal of mitochondria in cells. At the same time, however, mitochondrial mass becomes renewed by biogenesis. Depending on the balance between the mitophagy rate 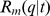 and the renewal rate 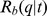, these process can add up to both a net loss or a net gain in quality state 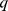. The combination of these processes is schematically shown in Fig. 1D and the corresponding master equation reads:
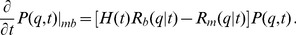
(7)Here, we introduced the dimensionless quantity
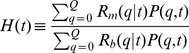
(8)that represents a homeostatic rheostat: The rate of mitochondrial biogenesis is continuously adapted until the net change of the total mitochondrial mass vanishes. This ensures that the norm Eq. (4) remains a constant in time, *i.e.* the total number of mitochondria in the cell is unchanged.

### Reference simulation of the mitochondrial life cycle

Combining Eqs. (5), (6) and (7) yields the master equation for the reference simulation of the mitochondrial life cycle:
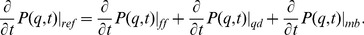
(9)This simulation accounts for fusion–fission events, functional quality decay and mitophagy in the presence of mitochondrial biogenesis under homeostatic conditions. It allows studying the impact of cycles of fusion and fission on the quality maintenance in the ensemble of mitochondria. Furthermore, it can be extended to account for molecular damage in aging cells.

### Mitochondrial life cycle simulation with molecular damage

**Figure 2:**
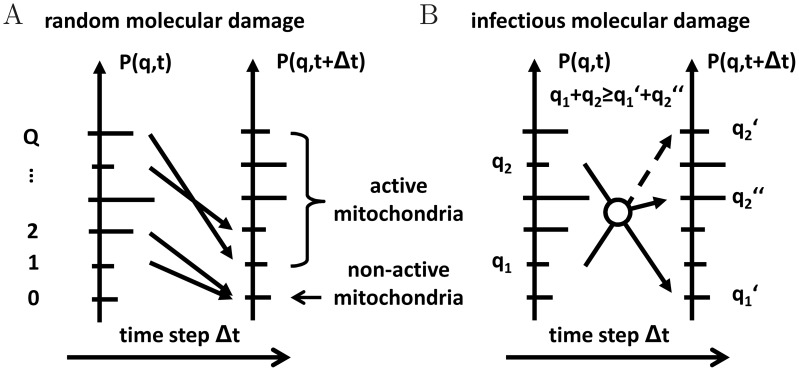
Schematic representation of the two types of molecular damage. *(A)* Random molecular damage, *e.g.* induced by ROS production, corresponds to a random re-distribution of 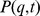 with the norm of 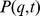 remaining constant at all times. *(B)* Infectious molecular damage is associated with the violation of the quality conservation during fusion–fission events resulting in a random decrease of the total quality from 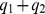 to 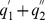.

We extend the reference simulation Eq. (9) by accounting for the impact of molecular damage in mitochondria that give rise to a decrease in their functional quality. As depicted in Fig. 2, molecular damage is assumed to be the result of two conceptually different types of damage: (i) random molecular damage and (ii) infectious molecular damage. Random molecular damage is caused by external or internal sources, *e.g.* by ROS, and occurs randomly in time and to a random degree concerning the severity of the damage. We use the term infectious molecular damage for impairments induced and/or propagated by cycles of fusion and fission. The inclusion of this type of damage was simulated for the following reasons. It was reported that a partial dissipation of the membrane potential occurred upon mitochondrial fission in mammalian cells [19]. This may also be accompanied by loss of mitochondrial ion homeostasis. In addition, certain types of molecular damage may exert a dominant-negative mode of function and thus any additional propagation by ongoing content mixing would impair mitochondrial function resembling an infection-like process. One example of such a dominant-negative-like behavior could relate to the possibility that certain mutant mtDNA molecules have a replicative advantage over wild type mtDNA molecules - a hypothesis that was proposed in a number of studies [43], [51], [52]. Consequently, mitochondria harboring wild type mtDNA molecules can only be outcompeted by the replicative advantage of mutant mtDNA molecules when they were infected by a fusion event with mitochondrial population harboring mutant mtDNA molecule. In this way a pre-existing mutation is spread over the entire network in a fusion-dependent manner. In our simulation, infectious molecular damage implies a violation of the quality conservation in the molecular exchange between mitochondria during fusion–fission events.

### Random molecular damage

The mitochondrial distribution in the quality state-space is affected by molecular damage induced by ROS production. As depicted in Fig. 2A, we consider this damage to occur with random impact on the mitochondrial distribution and the corresponding master equation may be written as:
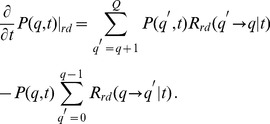
(10)Here, 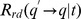 represents the random rate for leakage of the mitochondrial distribution from quality state 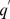 to 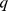. Note that this random process is restricted to values of 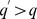 and enters Eq. (10) as the gain term. Similarly, the second term on the right-hand side of Eq. (10) represents the loss of mitochondria with functional quality 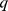 to quality states 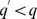.

Random molecular damage is considered to reshuffle the probability distribution while conserving its norm, which imposes constraints on the involved rates. In practice, we perform the computation in a way that ensures the norm conservation by construction. At each time step, we choose random pairs of quality states 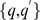 with 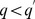 from a uniform distribution with the occurrence rate 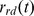 of damage. Next, we reduce the probability of the higher-quality state 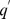 by a random fraction 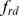 with 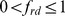 and simultaneously increase the probability of lower-quality state 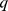 by this fraction:
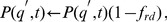
(11)

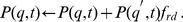
(12)Within the same time step, random pairs reshuffle 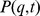 one after the other under conservation of the norm Eq. (4).

### Infectious molecular damage

This type of molecular damage is associated with the violation of the strict quality conservation condition 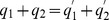 during fusion–fission events. As shown in Fig. 2B, two mitochondria in quality states 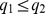 are fusing and subsequently undergoing fission into two mitochondria in quality states 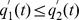. Thus, infectious molecular damage gives rise to a net loss of functional quality after fission, with the two mitochondria in quality states 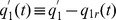 and 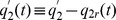, respectively. Here, 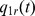 and 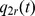 represent random values of a particular molecular exchange event and are drawn from a uniform distribution in the range 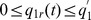 and 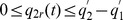.

In the probabilistic modeling approach, damage in the exchange of molecules between mitochondria is directly associated with fusion–fission events and occurs with rate 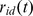. Note that this rate is limited by the fusion–fission rate 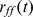 that corresponds to the maximal rate by which infectious molecular damage can occur. In practice, the list 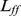 of all possible fusion–fission events is replaced by 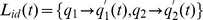, which has to be re-computed at each time step. Once 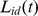 has been generated, we continue with the calculation of the corresponding master equation Eq. (5) on the basis of this list.

### Representation of transition rates

The processes of mitochondrial dynamics occur with characteristic transition rates that are specified within a product ansatz. For example, the fusion–fission rate involves the interaction of two mitochondria whose interaction may depend on the involved quality states and may change with time:
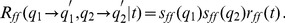
(13)Here, the rate 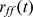 accounts for time-dependent changes of the fusion–fission events, while the selectivity function 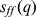 with 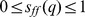 accounts for the involved quality state 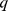, such that the occurrence rate of fusion–fission events is modulated by the functional quality of the involved mitochondria. Similarly, we represent the rates depending on a single mitochondrial quality level by
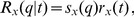
(14)where the index 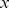 refers to the process of functional quality decay (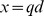), mitophagy (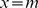), mitochondrial biogenesis (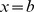), occurrence of random molecular damage (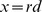), and occurrence of infectious molecular damage (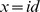).

The product ansatz provides full flexibility with regard to the dependence of the rates on the involved quality state 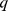 and the current time 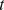. For example, the time-dependence may be specified by a Hill function:
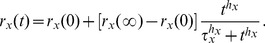
(15)Here, 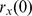 and 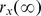 denote the initial (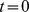) and final (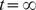) rate value, respectively. The monotonic transition between these two values is determined by the Hill exponent 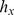 and the Hill coefficient 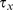, which corresponds to the time point at which the rate attains its half-value. For 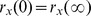 the rate is constant in time.

Note that the occurrence rate for infectious molecular damage, 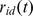, poses a special case. This rate is limited by the rate 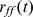 for the occurrence of fusion–fission events. Thus, the upper limit of the Hill function for 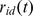 is set by the time-dependent fusion–fission rate, 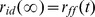, such that 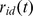 is not necessarily a monotonic function of time.

As is clear from Eqs. (13) and (14), the selectivity function modulates the time-dependence of the rate to account for the impact of quality state 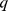 on the occurrence of the process. In analogy to the rates, the dependence of the selectivity functions on the quality state 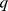 may as well be represented by Hill functions,
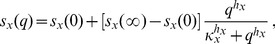
(16)where 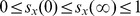, and 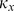 and 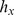 denote again the Hill coefficient and Hill exponent, respectively.

### Computer simulations and readout

We developed an algorithm for the time integration of the master equation with time step 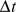. In all simulations the time step is chosen to be much smaller than the inverse of the largest rate and the stability of the time integration is checked by monitoring the conservation of the norm Eq. (4) at each time step. In all presented simulations, we obtained that 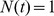 is preserved upto at least seven post decimal positions.

Furthermore, time-dependent changes in the probability distribution 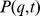 are monitored by computing the deviation factor
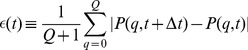
(17)at each time step. If the system reaches equilibrium, 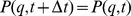, the deviation factor vanishes, 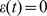, and the simulation may be stopped. All presented simulation results in the absence of random processes reached values for 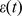 below 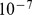.

It should be noted that the system reaches a flow equilibrium, where the involved processes – such as fusion–fission events, quality decay, mitophagy and biogenesis of mitochondrial mass – are continuously taking place but are balancing each other to yield 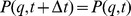.

In the presence of random processes, 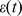 does not vanish but fluctuates around a constant average value that depends on the random strength of these processes. Thus, monitoring 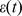 is still useful in order to check that a constant average value is reached in time. For all presented simulation results in the presence of random processes we typically observe values for 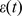 well below 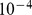, where averages of the characteristic quantities do not change anymore, indicating that the system has reached its quasi-equilibrium state.

The readout of the computer simulations includes the time-dependent fraction of mitochondria accumulating in quality state 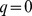 and being non-active:
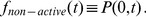
(18)The fraction of mitochondria remaining active is distributed over quality states 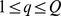 and calculated by summation:
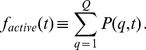
(19)While 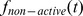 and 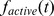 are quantitive measures of the relative size of active and non-active mitochondria populations in the cell, a qualitative measure is given by the time-dependent average quality. We compute the average quality of all mitochondria by summation over all quality states:
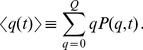
(20)However, since it turns out that the profile of 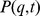 is typically characterized by a peak at state 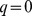 and a peak at high-quality states, the average quality over all states does not adequately represent this distribution. Therefore, we also compute the average quality of active mitochondria,
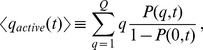
(21)involving only quality states 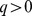 of active mitochondria.

## Results

### Cycles of fusion and fission ensure mitochondrial quality control

**Figure 3:**
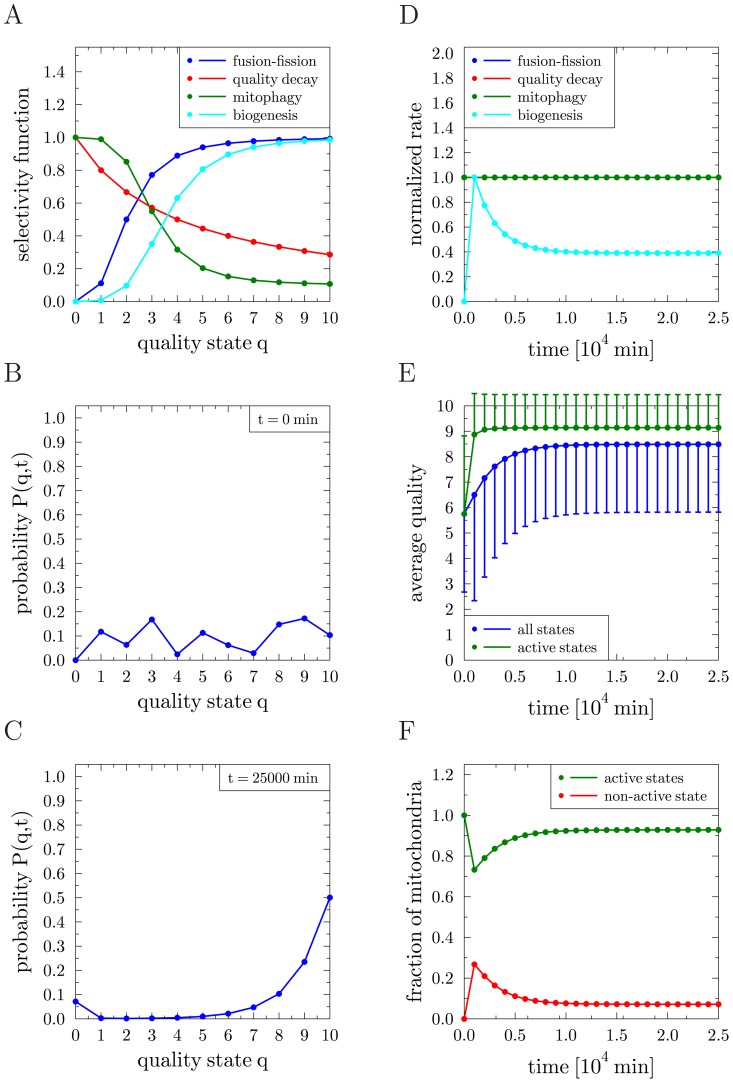
Results of the reference simulation. *(A)* Selectivity functions for all processes as function of quality 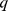. *(B)* Initial random distribution of 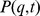 in quality state-space at time 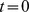 min. *(C)* Equilibrium distribution of 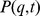 in quality state-space at time 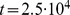 min. *(D)* Transition rates of all processes normalized to their individual maximal values as function of time. The blue, red and green curves are on top of each other. *(E)* Average quality of mitochondria as function of time over all states (blue) and over active states (green). Error bars correspond to the standard deviation of the distribution and are plotted single-sided for reasons of clarity. *(F)* Fraction of mitochondria in the non-active state (red) and in active states (green) as function of time.

To simulate mitochondrial quality control under standard conditions *in silico* we applied a probabilistic modeling approach taking into account well-known as well as novel aspects in mitochondrial biology. This simulation of the mitochondrial life cycle is defined by Eq. (9) and accounts for distinct quality states of mitochondria, fusion and fission events, for progressive decline of molecular integrity, and for mitochondrial turn-over (Fig. 1). All parameters of the reference simulation were either taken from the literature or were estimated (Table 1). The size of the quality state-space was chosen to be 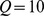 and the rates of all processes were set to constant values in time. In accordance with experimental findings [19], we set the time between two fusion–fission events to 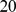 minutes, while the time scales on which mitochondrial quality decay and mitophagy take place were chosen to be orders of magnitude larger. This is in line with reports showing that turn-over of mitochondrial proteins in mammalian cells occurs with a half-life in the order of days [53]–[55]. The selectivity functions are represented by Hill functions according to Eq. (16) with the corresponding parameters given in Table 1. Although these parameters cannot directly be derived from experimental data, it is reasonable to expect that the selectivity functions resemble the qualitative behavior shown in Fig. 3A. Quality decay and mitophagy are gradually decreasing for increasing quality states, whereas mitochondrial biogenesis and fusion–fission events are both increasing functions of 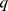. Note that the selectivity functions for mitochondrial mass renewal and fusion–fission events vanish at 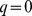, representing the fact that dysfunctional mitochondria are incapable of fusing with the intact mitochondrial network. Further, these mitochondria will undergo mitophagy with a higher probability than functional mitochondria. The simulation is started from a random distribution 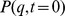 that is generated from a uniform distribution over 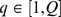 and is shown in Fig. 3B. At time 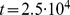 min it has evolved into the equilibrium distribution (Fig. 3C). Starting from different initial random distributions, we found that the reached equilibrium distribution in Fig. 3C is robust against these changes. Similarly, we confirmed that the qualitative simulation results do not depend on the choice of the size 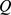 of the quality state-space and do not depend on the precise choice of the rate values as well as on the profile of the Hill functions Eq (15) and Eq (16), respectively, for the time-dependence and quality-dependence of the rates. Changes in these parameters merely affect kinetic aspects of the system, *e.g.* regarding the time scale on which the system equilibrates, and a discussion on this issue as well as on the impact of each process is provided by the Supporting Information.

**Table 1:** Parameters of the reference simulation.

Description	Symbol	Value
Quality state-space	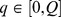	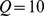
Fusion–fission rate	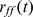	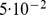 min^−1^
Fusion–fission selectivity	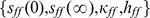	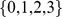
Quality decay rate	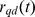	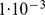 min^−1^
Quality decay selectivity	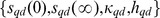	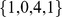
Mitophagy rate	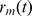	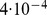 min^−1^
Mitophagy selectivity	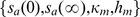	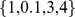
Biogenesis selectivity	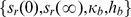	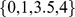
Time step	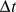	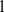 min

The parameter values were either estimated or adopted from Refs. [19], [53]–[55].

The simulations confirm that cycles of fusion and fission represent a reliable mechanism for mitochondrial quality enhancement and maintenance. The equilibrium distribution revealed that 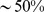 of all mitochondria in the cell reach the highest possible quality state 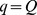, whereas only 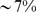 populate the state of fully dysfunctional mitochondria at 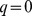. It should be noted that the equilibrium distribution represents a steady-state, where all processes are continuously taking place but are balancing each other. This is shown in Fig. 3D where we plot the rates of all processes normalized to their maximum value. All rates are constant in time, except for the renewal rate that is dynamically adapting under the imposed homeostatic conditions and attains the equilibrium value 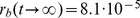 min^−1^. The time point at which the renewal rate becomes independent of time is an adequate measure for deciding that the system has reached its flow equilibrium. In Fig. 3E, we plot the average quality of mitochondria as a function of time, both over all quality states and over active states (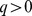) yielding 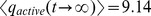. This number characterizes the equilibrium distribution of active mitochondria with the error bars reaching the size 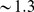 corresponding to the standard deviation of the distribution in Fig. 3C for 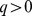. Note that, since the typical profile of the equilibrium distribution is double-peaked at state 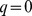 and at high-quality states, interpretation of the average quality over all states may be misleading, which is why we also present the average quality restricted to active mitochondria.

The proper functioning of the cell will require mitochondria of sufficiently high quality to be present at sufficiently high quantities. As can be seen from Fig. 3F, in addition to the fact that the average quality of active mitochondria is high, the fraction of active mitochondria reaches the high value of 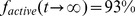. Taken together, it can be concluded that cycles of fusion and fission combined with selective removal of dysfunctional mitochondria give rise to large amounts of active mitochondria with high average quality. All subsequent simulations are started from the equilibrium distribution of the reference simulation (see Fig. 3C) as initial distribution.

### Random molecular damage is predicted to have a major effect on mitochondrial morphology

**Figure 4:**
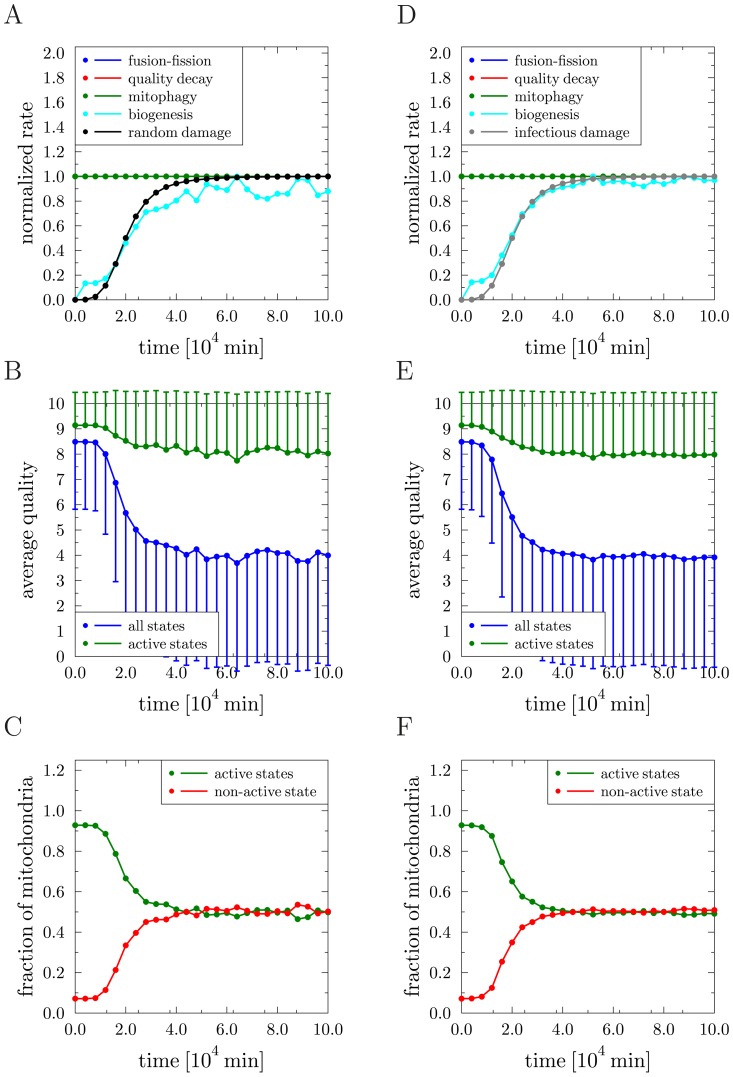
Results of the reference simulation in the presence of molecular damage. *(A)*–*(C)* Random molecular damage: *(A)* Transition rates of all processes normalized to their individual maximal values as function of time. The blue, red and green curves are on top of each other. *(B)* Average quality of mitochondria as function of time over all states (blue) and over active states (green). Error bars correspond to the standard deviation of the distribution and are plotted single-sided for reasons of clarity. *(C)* Fraction of mitochondria in the non-active state (red) and in active states (green) as function of time. *(D)*–*(F)* Infectious molecular damage: the same quantities as in *(A)*–*(C)* are plotted.

Next we decided to address whether and how such a system can handle the appearance of random molecular damage *e.g.* by ROS. In the presence of random molecular damage, as depicted in Fig. 2A, the master equation reads
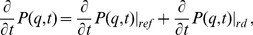
(22)where the individual terms are given by Eqs. (9) and (10), respectively. Starting computer simulations from the equilibrium configuration of the reference simulation (see Fig. 3C), all parameters of the reference simulation were left unchanged (see Table 1). In addition, the occurrence rate of random molecular damage follows a Hill function with parameter values 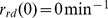, 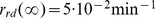, 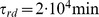, 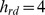, and the random fraction is set to 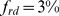 (see Eqs. (11) and (12)). In Fig. 4A the normalized rates are plotted as a function of time. The renewal rate was again observed to dynamically adapt under the imposed homeostatic conditions. However, due to the presence of random molecular damage, its value was fluctuating and attained the constant average value 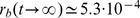 min^−1^. As is observed in Fig. 4B, the quality of active mitochondria was decreasing to the average value 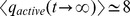 with increased standard deviation of 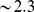. Thus, compared to the reference simulation, in the presence of random molecular damage the quasi-equilibrium distribution became broader and had a lower average quality of active mitochondria. However, in contrast to this modest change in the average quality of active mitochondria, a significant difference was observed for the fraction of active mitochondria in the cell under steady-state conditions (Fig. 4C). This value was decreased by 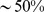 compared to the reference simulation indicating that random molecular damage can give rise to an inversion: While in the reference simulation 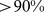 of all mitochondria were active and occupied high-quality states, random molecular damage caused a reduction to about of only 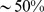 of all mitochondria to accumulate in the highest quality state. Conversely, the number of dysfunctional mitochondria with the lowest quality state (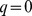) increased drastically from about 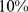 in the reference simulation to 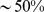 here (Fig. 4C). As dysfunctional mitochondria are modeled to be non-fusogenic, which is consistent with experimental data obtained for mammalian cells [16], [56], our simulations imply that the mitochondrial network becomes fragmented. Taken together, upon random molecular damage we observe the emergence of two major classes of mitochondria: one class characterized by high average quality actively undergoing fusion and fission cycles, and one class of non-active and non-fusogenic mitochondria. Thus, mitochondrial morphology is a good parameter for the relative abundance of these two major classes.

### Infectious and random molecular damage show similar effects on mitochondrial quality

Intrigued by the observation that following mitochondrial fission the mitochondrial membrane potential was diminished in a fraction of the resulting daughter mitochondria [19] we decided to test what implications any fusion–fission dependent damage would have on mitochondrial quality control. We termed this ‘infectious molecular damage’ as it may not only inflict damage directly caused by fission events but, in addition, it may also represent loss of mitochondrial ion homeostasis, or a propagation of dominant-negative properties (*e.g.*, spreading of mutant mtDNA with a replicative advantage over wild type mtDNA) by ongoing content mixing. Therefore, we extended the reference simulation by the addition of infectious molecular damage induced by fusion–fission events. The corresponding master equation reads
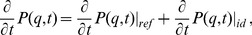
(23)where the individual terms are given by Eqs. (9) and (5) with random list 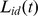 instead of 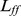. The computer simulation is again started from the equilibrium configuration of the reference simulation (see Fig. 3C) and all parameters of the reference simulation remain unchanged (see Table 1). In addition, the occurrence rate of infectious molecular damage follows a Hill function with parameter values 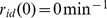, 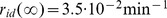, 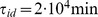, and 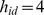. In Fig. 4D the normalized rates are plotted as a function of time, where the renewal rate reaches the constant average value 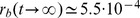 min^−1^. For the two types of molecular damage, the resulting mitochondria distributions were strikingly similar (Fig. 4E and F). In fact, without knowledge about the underlying damaging mechanisms it was not possible to distinguish them when comparing Figs. 4B and C with Figs. 4E and F. Taken together, for both types of damage the statement that a modest decrease of the average quality of active mitochondria can be accompanied by a significant decrease in the fraction of active mitochondria remains valid.

### Decelerating mitochondrial dynamics during aging improves mitochondrial quality control

**Figure 5:**
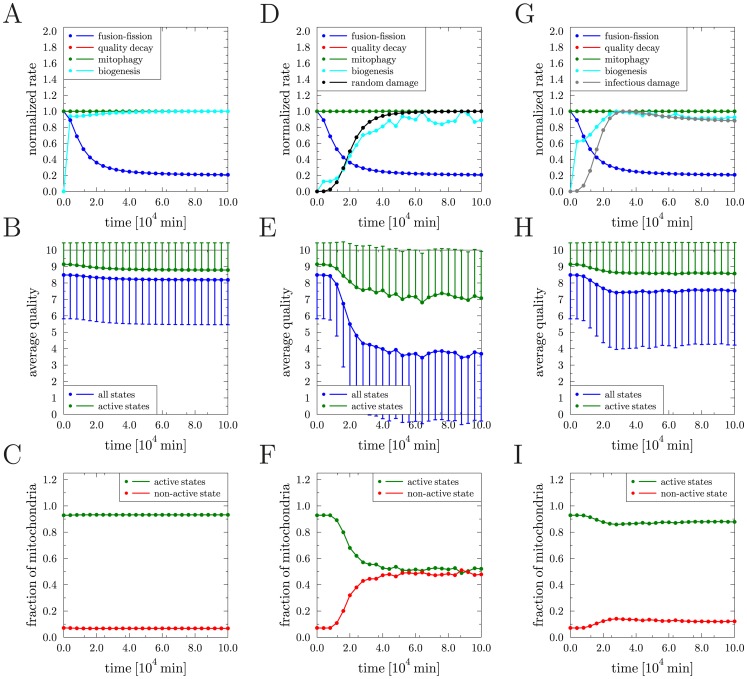
Simulation results for implementing a time-dependent adaptation of the fusion–fission rate. *(A)*–*(C)* Reference simulation: *(A)* Transition rates of all processes normalized to their individual maximal values as function of time. The blue, red and green curves are on top of each other. *(B)* Average quality of mitochondria as function of time over all states (blue) and over active states (green). Error bars correspond to the standard deviation of the distribution and are plotted single-sided for reasons of clarity. *(C)* Fraction of mitochondria in the non-active state (red) and in active states (green) as function of time. *(D)*–*(F)* Random molecular damage: the same quantities as in *(A)*–*(C)* are plotted. *(G)*–*(I)* Infectious molecular damage: the same quantities as in *(A)*–*(C)* are plotted.

Since aging of cells was associated with a decrease in the fusion–fission rate, we investigated the impact of reducing this rate in the course of time. We perform and compare simulations for the following three cases: (i) reference simulation without molecular damage, (ii) reference simulation with random molecular damage, and (iii) reference simulation with infectious molecular damage during fusion–fission events. The same parameter sets as described before for the individual simulations were used. Only, the dynamics of the fusion–fission rate 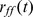 was altered as follows. In all three cases it follows a Hill function with parameters 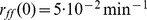, 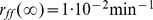, 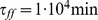, and 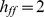 resulting in a progressive reduction to about 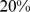 of the initial fusion–fission rate within 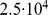 minutes. The results are summarized in Fig. 5. The normalized rates for the three cases are presented in Figs. 5A, D, and G and the renewal rates attain the average values 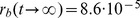 min^−1^ in the absence of molecular damage, 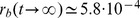 min^−1^ for random molecular damage, and 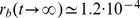 min^−1^ for infectious molecular damage. In the absence of molecular damage and for decreasing fusion–fission rate, we found that both the average quality of active mitochondria (see Fig. 5B) as well as the fraction of active and non-active mitochondria (see Fig. 5C) were virtually unchanged. The situation changed in the presence of random molecular damage. In this case, decreasing the fusion–fission rate had a visible impact on the average quality of active mitochondria, as observed by comparing Fig. 5E with Fig. 4B for constant fusion–fission rate: The average quality value was decreased to 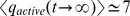 while the standard deviation was increased to 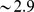. This result is a consequence of the fact that cycles of fusion and fission maintain the mitochondrial quality and decreasing the occurrence rate of fusion–fission events renders the system more susceptible to the random molecular damage. Note that the fractions of active and non-active mitochondria presented in Fig. 5F remained fairly unchanged when compared to Fig. 4C for constant fusion–fission rate. The situation is very different for infectious molecular damage induced by fusion–fission events. In this case, reducing the rate of fusion–fission events averted infectious molecular damage because their occurrence is limited by the fusion–fission rate itself. This is seen by comparing the rate dynamics in Fig. 5G with Fig. 4D for constant fusion–fission rate. In the present case, the rate of infectious molecular damage is confined to 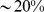 of its maximum value for constant fusion–fission rate. As a direct consequence, the average quality of active mitochondria in Fig. 5H was found to be preserved at values close to the average quality in the absence of molecular damage (see Fig. 5B). The difference to the situation simulating random molecular damage was even more pronounced by comparing the fractions of active and non-active mitochondria in Figs. 5F and I. Reducing the fusion–fission rate ensured that more than 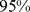 of all mitochondria remained active, a value that is close to the reference simulation without molecular damage (see Fig. 5C). Taken together, the simulation results suggest that, if molecular damage is predominantly induced and propagated by fusion–fission events, then it is favorable for the quality control system to reduce the occurrence of fusion–fission events itself. In other words, if quality maintenance by fusion–fission events cannot be guaranteed to be performed without damage, then it may be of advantage for the system to abstain from this process altogether.

**Figure 6:**
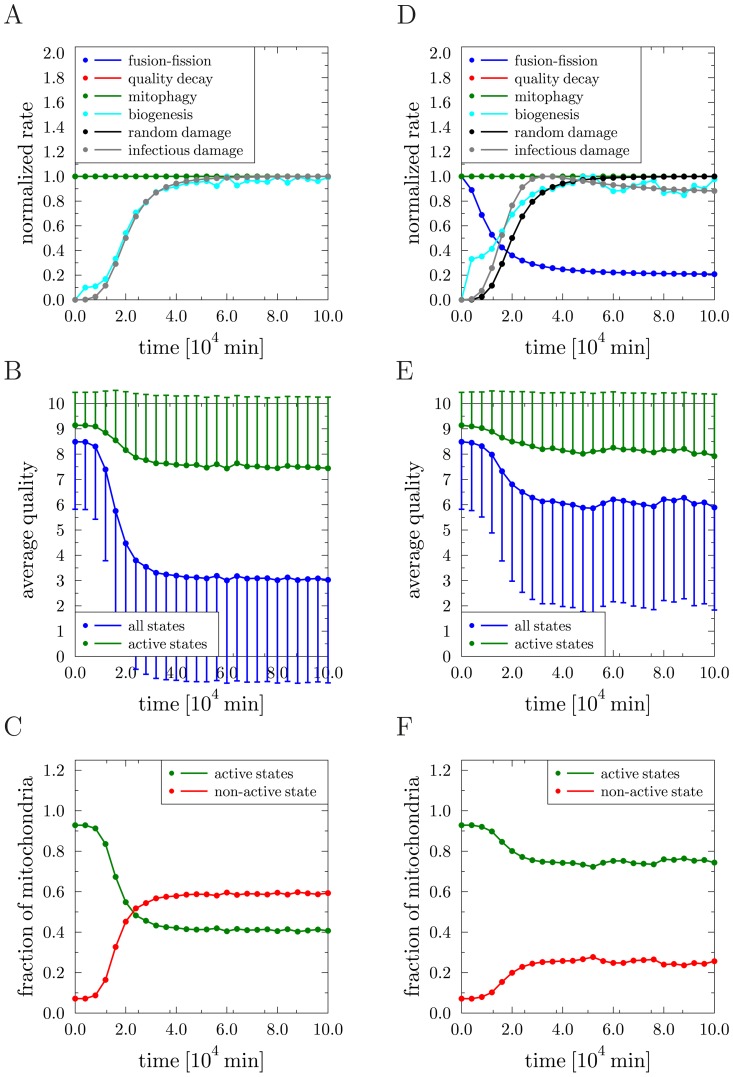
Results of the reference simulation in the presence of molecular damage. *(A)*–*(C)* Random molecular damage triggering infectious molecular damage at a constant fusion–fission rate: *(A)* Transition rates of all processes normalized to their individual maximal values as function of time. The blue, red and green curves as well as the black and grey curves are on top of each other. *(B)* Average quality of mitochondria as function of time over all states (blue) and over active states (green). Error bars correspond to the standard deviation of the distribution and are plotted single-sided for reasons of clarity. *(C)* Fraction of mitochondria in the non-active state (red) and in active states (green) as function of time. *(D)*–*(F)* Random molecular damage triggering infectious molecular damage with time-dependent fusion–fission rate: the same quantities as in *(A)*–*(C)* are plotted.

The above considerations motivated us to test a scenario in which random molecular damage, *e.g.* as a consequence of ROS production in mitochondria of aging cells, is propagated via fusion–fission events. This mechanism can be regarded as an infection-like process, *e.g.* spreading a dominant-negative trait to neighboring mitochondria and thereby enhancing the overall damage further. In the simulation we account for the coupling of these two mechanisms by the master equation
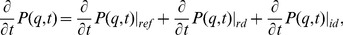
(24)where the occurrence rate 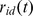 of infectious molecular damage is directly related to the occurrence rate 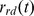 of random molecular damage induced by ROS production. That is, the frequency of infectious molecular damage is assumed to be controlled by two factors: (i) the requirement that random molecular damage occurs with rate 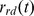, and (ii) the limitation as set by the occurrence rate 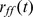 of fusion–fission events. This was taken into account by the ansatz
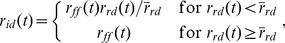
(25)where the constant rate 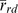 denotes the limiting value of 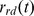 above which 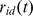 attains the maximal value 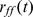. Simulation results are presented in Fig. 6, where we compare again the case of a constant and a time-dependent fusion–fission rate using the same parameters as in the simulations presented in Fig. 5 and setting 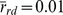 min^−1^. The normalized rates are presented in Figs. 6A and D, where the renewal rate attains the values 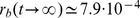 min^−1^ and 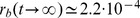 min^−1^, respectively, for the case of constant and of time-dependent fusion–fission rate. We observed for constant fusion–fission rate that the combination of random molecular damage with infectious molecular damage during fusion–fission events gave rise to an additional decrease in the average quality of active mitochondria (Fig. 6B) as compared to the cases with only one type of molecular damage (see Figs. 4B and E). The fraction of non-active mitochondria is raised from 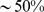 in Figs. 4C and F to 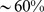 in Fig. 6C. While these changes could be expected, we observed that the decrease of the fusion–fission rate with time gives rise to a significant improvement of the systemic behavior. In Fig. 6E it is shown that the average quality of active mitochondria is maintained at previous levels (see Figs. 4B and E) and the fraction of active mitochondria is increased from 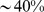 for constant fusion–fission rate (Fig. 6C) to 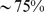 (Fig. 6F). These results suggest that a reduction of the fusion–fission rate in the cellular ensemble of mitochondria may be generally viewed to function as a systemic escape mechanism that enables a cellular lifespan extension.

## Discussion

During the past years of research mitochondria turned out to be highly dynamic organelles which are constantly in the process of fission and fusion. These processes bear important consequences explaining the roles of mitochondrial pathways involved in processes leading to mitochondrial impairments and a number of diseases and in aging. Our simulations confirm the current view that cycles of fusion and fission and mitophagy represent a reliable and robust mechanism of maintaining functional mitochondria.

In fact, the computer simulations show that this mechanism does not only maintain a functional and dynamic network of mitochondria but is also capable of driving the system into this state. Moreover, our study provides some unexpected and conceptually important findings regarding the role of mitochondrial dynamics during ageing. We propose that a decrease of the fusion-fission rate as a sign of growing old may actually be viewed in a positive way, namely as the adaptation of this complex biological system to counteract the fate of dying young. The basis for our model which we termed ‘mitochondrial infectious damage adaptation’ (MIDA) model is explained in detail below.

To study the impact of aging processes on mitochondrial quality control, we consider two different types of molecular damage which we termed: (i) ‘random molecular damage’ generated by ROS or by other processes impairing the molecular function of constituents (*e.g.* lipids, proteins, DNA) of mitochondria, and (ii) ‘infectious molecular damage’ occurring during the molecular exchange between mitochondria via fusion–fission events. Random molecular damage is well documented to occur and a vast literature demonstrates that it is implicated in the aging process (for reviews see [6],[10]–[12]). The nature of ‘infectious molecular damage’ is less understood but is well documented to exist as, *e.g.* a partial dissipation of the mitochondrial membrane potential was observed to occur upon fission of mammalian mitochondria [19]. On top, this may be accompanied by loss of mitochondrial ion homeostasis, or the spreading of a dominant-negative factor such as mutant mtDNA harboring a replicative advantage over wild type mtDNA. These two types of molecular damage have in common that they give rise to a functional impairment of mitochondria which is well reflected by our computer simulations. Even though the probabilistic modeling approach does not directly simulate mitochondrial morphology it implies that for both types of molecular damage fragmentation of the mitochondrial network is increased which is fully in line with experimental observations [16], [37], [56]. We predict that upon molecular damage two major classes of mitochondria are generated: one class characterized by high average quality actively undergoing fusion and fission cycles, and one class of non-active and non-fusogenic mitochondria. This is of practical importance as changes in the morphology of the mitochondrial network represent a sensitive measure that can be directly and easily determined experimentally by fluorescence microscopy.

The process of aging is typically occupied with negative associations. Similarly, it is tempting to readily associate the experimentally observed decrease in the fusion–fission rate of mitochondria in aging cells [47] with the interpretation that this decrease may be a cause of aging itself. However, this conclusion is called into question by our simulations. Decreasing the fusion–fission rate implies a reduction in the enhancement and maintenance of the functional quality of mitochondria. However, the resulting impairment of mitochondrial quality control is less pronounced when content mixing between mitochondria is performed under the assumption that infectious molecular damage occurs. The latter type of damage acts as a mean to propagate already existing damage and thus it is favorable to decelerate fusion and fission cycles of mitochondria in order to diminish its damage propagating consequences. By that a larger fraction of active mitochondria is maintained in the system for a longer period of time at the acceptable costs of a minor decrease in their average quality. However, the system becomes more vulnerable to additional random molecular damage since dysfunctional mitochondria are less efficiently eliminated from the mitochondrial network. Taken together, based on recent experimental findings and our simulations shown here, we reason that the quality decay of mitochondria is promoted by two distinct types of damage, random and infectious molecular damage, and that both types of damage are linked.

**Figure 7:**
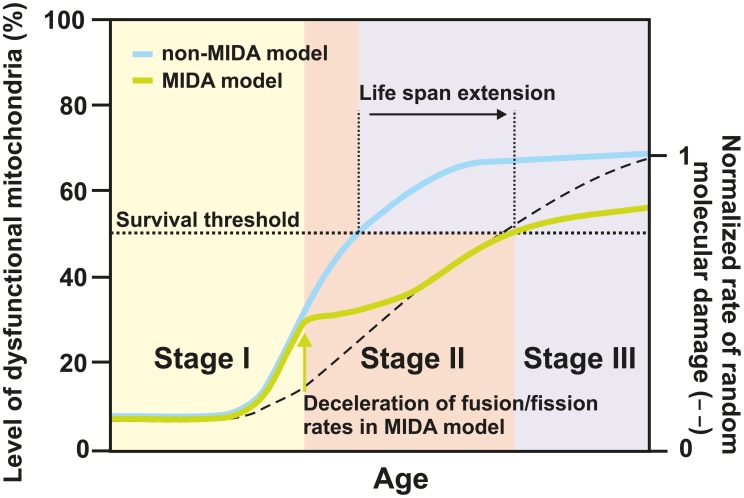
Mitochondrial infectious damage adaptation (MIDA) model. Schematic representation showing the loss of mitochondrial quality over the fictive life span of an organism according to the MIDA model (green) versus a non-MIDA model (blue). In the MIDA model fusion–fission rates are reduced when a certain degree of molecular damage has accumulated (green arrow). In the non-MIDA model this adaptation was omitted. Stage I is characterized by high fusion–fission rates, low levels of accumulated random molecular damage, yet a high removal rate of those few dysfunctional mitochondria. Stage II represents the time/age when already a significant amount of molecular damage has accumulated. This damage is propagated and enhanced by ongoing fusion and fission cycles, representing a distinct type of damage, termed ‘infectious molecular damage’. At some time point the latter outweighs the benefit of mitochondrial dynamics and mitophagy in removing dysfunctional mitochondria. Decelerating mitochondrial dynamics (green arrow), in the MIDA model, slows down the accumulation of dysfunctional mitochondria compared to the situation in the non-MIDA model. Still, this adaptation in the MIDA model renders the system less capable of dealing with additional random molecular damage. Assuming a certain survival threshold (dotted line) this results in a net life span extension. Reaching this threshold marks stage III and cell death. The rates for mitophagy, quality decay, and mitochondrial biogenesis under homeostatic conditions are kept constant over all stages. The full simulation and used parameters are provided in the Supporting Information.

The scenario outlined above prompts us to propose the MIDA model (see Fig. 7 and the Supporting Information). It considers the possibility of inter-mitochondrial infection by molecular damage and may be divided into three different stages during lifetime. At first, molecular damage occurs randomly by which the functional quality of an initially relatively small fraction of mitochondria becomes reduced. Nevertheless, even a small fraction of randomly damaged mitochondria can have severe consequences when this damage is propagated by cycles of fusion and fission. However, this damage is not only propagated but even enhanced as fusion and fission themselves cause another type of damage, which we termed ‘infectious molecular damage’. We suggest that mitochondria that have been randomly damaged may be held responsible for infectious molecular damage in subsequent fusion–fission events. Once the overall molecular damage gets established in the mitochondrial network the associated loss in functional quality of mitochondria and in the function of the whole cell is assumed to be counteracted by a systemic response. This marks the second stage of the mitochondrial infection scenario, where the rate of fusion–fission events is adapting to lower values in order to limit spreading of molecular damage in the cellular system. How this is regulated mechanistically is unclear but it is consistent with a study reporting that during cellular aging fusion and fission rates are strongly reduced [47]. Rather than viewing this reduction as a main cause of aging, this may actually be a systemic response to prolong cellular functioning. Finally, the third stage is characterized by increased ROS production in aging cells as observed in numerous systems during aging [6]–[12], [34]. The adapted system with decreased fusion–fission rate, however, is now in a more vulnerable state less capable to cope with this situation. While the impact of random molecular damage may be significantly delayed by the adaptation of the system in stage two, this kind of damage will ultimately gain the upper hand, enforce the system transition into stage three, and finally cause cell death. We predict that the capability to get rid of dysfunctional mitochondria would be even further reduced when in addition also the rate of mitophagy is decreased. The latter is not unreasonable as it was reported that autophagy is downregulated during normal aging (for review see [57]).

The MIDA model introduced here does well accommodate previous experimental data that have been generated in the past demonstrating a fundamental role of mitochondria in biological aging which, at that time, could not be interpreted in the view of constantly ongoing mitochondrial fission and fusion events. In particular, the accumulation of defective mtDNA as the result of initial damaging processes is well documented to occur during aging of biological systems from fungal aging models up to humans [58]–[67]. These processes may result from ROS induced damage, *e.g.* single point mutations or gross mtDNA rearrangement. The latter may be the result of the activity of autonomous DNA elements (*e.g.*, circular and linear plasmids in fungi) acting as ‘mutators’ or occur during mtDNA replication [62], [68]–[73]. Some of the corresponding processes have been linked to ‘infectious agents’ [74] and the transmission of a senescent stage from old to young individuals. Significantly, in these processes single events lead to the generation of defective mtDNAs which become suppressive and replace the unmutated mtDNA molecules. In mammals, this process is termed ‘clonal expansion’ [44]–[46]. After the abundance of mutated mtDNAs passes certain thresholds physiological consequences like bioenergetic impairments as they are characteristic for aging occur. As a response, retrograde signaling to the nucleus leads to alterations in gene expression and readjustments in metabolism [75], [76].

Defective mtDNA molecules arising during aging in the different systems behave like the postulated dominant-negative factors which are mixed and spread during mitochondrial fusion–fission cycles. The reduction in mitochondrial fusion–fission rates observed during normal aging in cell cultures appears to be an adaptive effort to limit the spread of such factors. Systems where such processes appear to be up-regulated, like in wildtype cultures of *P. anserina*, where an age-related increase in transcripts of the gene coding for the Dnm1 fission factor has been demonstrated, are short-lived. Affecting the genetic system which controls mitochondrial fission, *e.g.* via deletion of Dnm1 leads to an increase in life span [34]. In fact, the conclusions which are mainly derived from experimental data from studies with lower aging model organisms or mammalian cell cultures generate predictions that can be tested in the future also in higher organisms.

To test whether deceleration of fusion–fission cycles prolongs life span in mammals one could downregulate fusion and fission factors in a mammalian ageing model such as the mutator mouse. These mice express an error-prone mitochondrial 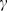-DNA-polymerase, leading to a drastic increase in the number of mtDNA mutations, mitochondrial dysfunction, different ageing-related phenotypes, and a significant lifespan shortening [77], [78]. As non-conditional deletions of genes for fusion or fission components are embryonic lethal in mice [79]–[82] one would need to downregulate one or more of these factors in a conditional manner using *e.g.* an inducible promoter. Controlled lowering of fusion and fission in the mutator mouse with increased molecular damage could be expected to reduce spreading of damage and thus may revert the accelerated aging phenotype at least partially.

Although our study clearly focuses on the role of mitochondrial dynamics in the ageing process it has the potential to challenge our current view on mitochondrial quality control in general. Changes in fusion–fission dynamics were recently observed under different physiological situations. After starvation or under certain stress conditions mitochondria were reported to undergo hyperfusion due to reduced DRP1-dependent mitochondrial fission [83]–[85]. Mitochondrial hyperfusion after starvation was not only shown for several cell lines but also for primary mouse hepatocytes [84]. Also progression from G1 to S phase requires hyperfusion of mitochondria whereas mitochondria fragment prior to cell division [40]–[42]. From these studies one can safely predict that fusion–fission rates are strongly dependent on the type of tissue (whether it is post-mitotic such as neurons or not) and on the metabolic state. Still, one needs to emphasize that there are hardly any quantitative data from primary tissues available. Based on this shortage of fundamental data, we feel that it is important to consider the possibility that mitochondrial content mixing by fusion–fission events can have beneficial as well as detrimental effects. The parameters when one or the other is dominating *in vivo* still need to be determined.

Overall, considering contradicting and counterintuitive data raised over the past decade of research on mitochondria and using these data in a mathematical modeling approach has led us to suggest a novel adaptive role of the molecular processes which control mitochondrial dynamics. The MIDA model does accommodate many of the poorly understood observations obtained over half a century of work on the biology of mitochondria and their role in aging. Although specific processes (*i.e.*, the mechanisms of amplification of defective mtDNA molecules) are still not finally solved, the model is an important step forward to understand the spreading of rare defective mtDNAs as it is found to occur during aging in biological systems from fungi to humans. The relevance of mitochondrial dynamics for the aging process and the evolution of the mitochondrial genome has recently been addressed in the ‘organelle control’ theory [49]. Central aspects addressed in this study were the evolution of mitochondrial fusion and fission and how these processes are related to the accumulation of mitochondrial mutants. While also addressing the accumulation and spreading of mutated mtDNA and the relation to mitochondrial dynamics, the MIDA model incorporates additional processes of mitochondrial quality control like mitophagy and ‘infectious molecular damage’. The predictions made by the MIDA model, *e.g.* about the impact of the deceleration of mitochondrial dynamics of organismal aging in general, will help to design experiments for testing it, to initiate refinements/extensions of the model, and in the long run to better understand mitochondrial quality control itself.

## Supporting Information

Figure S1 Flow chart of the program developed to perform the time integration of the master equation. Equation numbers refer to the main text.
(TIF)

Figure S2Results of the reference simulation in the absence of fusion–fission events. *(A)* Selectivity functions for all processes as function of quality 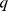. *(B)* Initial random distribution of 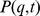 in quality state-space at time 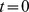 min. *(C)* Probability distribution 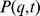 in quality state-space at time 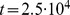 min. *(D)* Transition rates of all processes normalized to their individual maximal values as function of time. The red and green curves are on top of each other. *(E)* Average quality of mitochondria as function of time over all states (blue) and over active states (green). Error bars correspond to the standard deviation of the distribution and are plotted single-sided for reasons of clarity. *(F)* Fraction of mitochondria in the non-active state (red) and in active states (green) as function of time.(TIF)

Figure S3Results of the reference simulation in the absence of quality decay. *(A)* Selectivity functions for all processes as function of quality 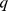. *(B)* Initial distribution of 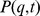 in quality state-space at time 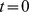 min. *(C)* Equilibrium distribution of 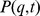 in quality state-space at time 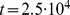 min. *(D)* Transition rates of all processes normalized to their individual maximal values as function of time. The blue and green curves are on top of each other. *(E)* Average quality of mitochondria as function of time over all states (blue) and over active states (green). Error bars correspond to the standard deviation of the distribution and are plotted single-sided for reasons of clarity. *(F)* Fraction of mitochondria in the non-active state (red) and in active states (green) as function of time.(TIF)

Figure S4Results of the reference simulation in the absence of mitophagy and mitochondrial biogenesis. *(A)* Selectivity functions for all processes as function of quality 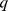. *(B)* Initial random distribution of 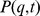 in quality state-space at time 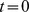 min. *(C)* Probability distribution 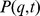 in quality state-space at time 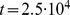 min. *(D)* Transition rates of all processes normalized to their individual maximal values as function of time. The blue and red curves are on top of each other. *(E)* Average quality of mitochondria as function of time over all states (blue) and over active states (green). Error bars correspond to the standard deviation of the distribution and are plotted single-sided for reasons of clarity. *(F)* Fraction of mitochondria in the non-active state (red) and in active states (green) as function of time.(TIF)

Figure S5Results of the reference simulation in the absence of fusion–fission events, mitophagy and mitochondrial biogenesis. *(A)* Selectivity functions for all processes as function of quality 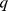. *(B)* Initial random distribution of 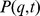 in quality state-space at time 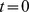 min. *(C)* Probability distribution 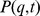 in quality state-space at time 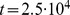 min. *(D)* Transition rates of all processes normalized to their individual maximal values as function of time. *(E)* Average quality of mitochondria as function of time over all states (blue) and over active states (green). Error bars correspond to the standard deviation of the distribution and are plotted single-sided for reasons of clarity. *(F)* Fraction of mitochondria in the non-active state (red) and in active states (green) as function of time.(TIF)

Figure S6Results of the reference simulation in the absence of fusion–fission events and quality decay. *(A)* Selectivity functions for all processes as function of quality 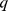. *(B)* Initial random distribution of 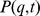 in quality state-space at time 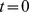 min. *(C)* Probability distribution 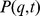 in quality state-space at time 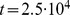 min. *(D)* Transition rates of all processes normalized to their individual maximal values as function of time. *(E)* Average quality of mitochondria as function of time over all states (blue) and over active states (green). Error bars correspond to the standard deviation of the distribution and are plotted single-sided for reasons of clarity. *(F)* Fraction of mitochondria in the non-active state (red) and in active states (green) as function of time.(TIF)

Figure S7Results of the reference simulation in the absence of quality decay, mitophagy and mitochondrial biogenesis. *(A)* Selectivity functions for all processes as function of quality 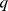. *(B)* Initial random distribution of 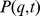 in quality state-space at time 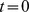 min. *(C)* Equilibrium distribution of 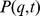 in quality state-space at time 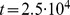 min. *(D)* Transition rates of all processes normalized to their individual maximal values as function of time. *(E)* Average quality of mitochondria as function of time over all states (blue) and over active states (green). Error bars correspond to the standard deviation of the distribution and are plotted single-sided for reasons of clarity. *(F)* Fraction of mitochondria in the non-active state (red) and in active states (green) as function of time.(TIF)

Figure S8Results of the reference simulation with altered set of selectivity functions. *(A)* Selectivity functions for all processes as function of quality 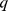. The blue, red and cyan curves are on top of each other. *(B)* Initial random distribution of 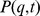 in quality state-space at time 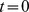 min. *(C)* Equilibrium distribution of 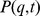 in quality state-space at time 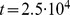 min. *(D)* Transition rates of all processes normalized to their individual maximal values as function of time. The blue, red and green curves are on top of each other. *(E)* Average quality of mitochondria as function of time over all states (blue) and over active states (green). Error bars correspond to the standard deviation of the distribution and are plotted single-sided for reasons of clarity. *(F)* Fraction of mitochondria in the non-active state (red) and in active states (green) as function of time.(TIF)

Figure S9 Results of the reference simulation in the presence of molecular damage as in Fig. 4 of the main text but for time-pulsed damage rates. *(A)*–*(C)* Random molecular damage: *(A)* Transition rates of all processes normalized to their individual maximal values as function of time. The blue, red and green curves are on top of each other. *(B)* Average quality of mitochondria as function of time over all states (blue) and over active states (green). Error bars correspond to the standard deviation of the distribution and are plotted single-sided for reasons of clarity. *(C)* Fraction of mitochondria in the non-active state (red) and in active states (green) as function of time. *(D)*–*(F)* Infectious molecular damage: the same quantities as in *(A)*–*(C)* are plotted.
(TIF)

Figure S10 Simulation results of the MIDA model. *(A)*–*(C)* Random molecular damage triggering infectious molecular damage at a constant fusion–fission rate: *(A)* Transition rates of all processes normalized to their individual maximal values as function of time. The blue, red and green curves are on top of each other. *(B)* Average quality of mitochondria as function of time over all states (blue) and over active states (green). Error bars correspond to the standard deviation of the distribution and are plotted single-sided for reasons of clarity. *(C)* Fraction of mitochondria in the non-active state (red) and in active states (green) as function of time. *(D)*–*(F)* Random molecular damage triggering infectious molecular damage with time-dependent fusion–fission rate: the same quantities as in *(A)*–*(C)* are plotted.
(TIF)

Table S1 Overview of results for the reference simulation with varied parameters.
(TIF)

Text S1 Supporting information on the algorithm and additional computer simulations.
(PDF)
