# Supplementary figures and images for: Deceleration of Fusion–Fission Cycles Improves Mitochondrial Quality Control during Aging
Source: PLoS Comput Biol. 2012 Jun 28;8(6):e1002576. doi: 10.1371/journal.pcbi.1002576 (PMC3386171; doi:10.1371/journal.pcbi.1002576)

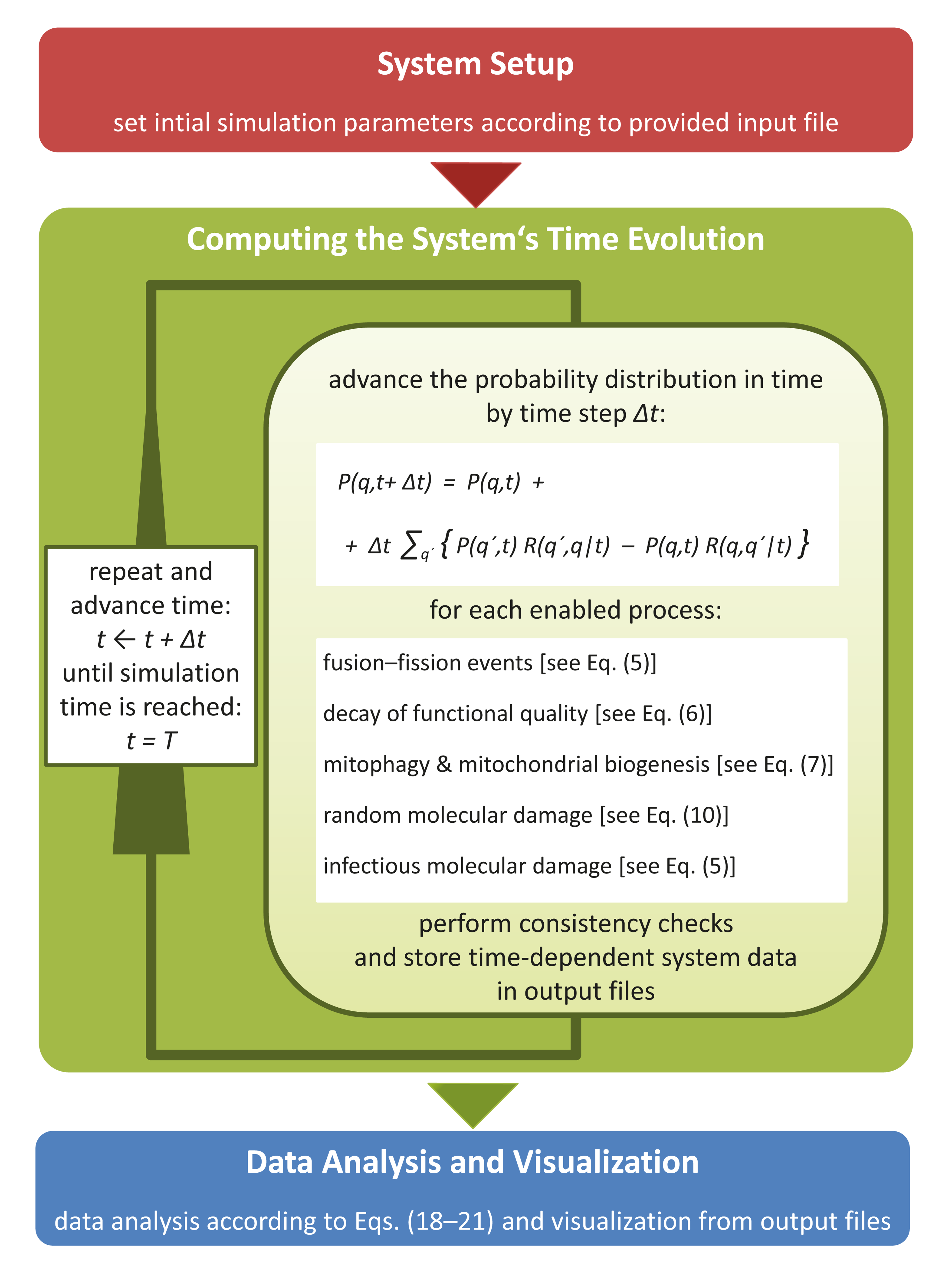

Supplement: Figure S1 — Flow chart of the program developed to perform the time integration of the master equation. Equation numbers refer to the main text. (TIF) [file pcbi.1002576.s001.tif]

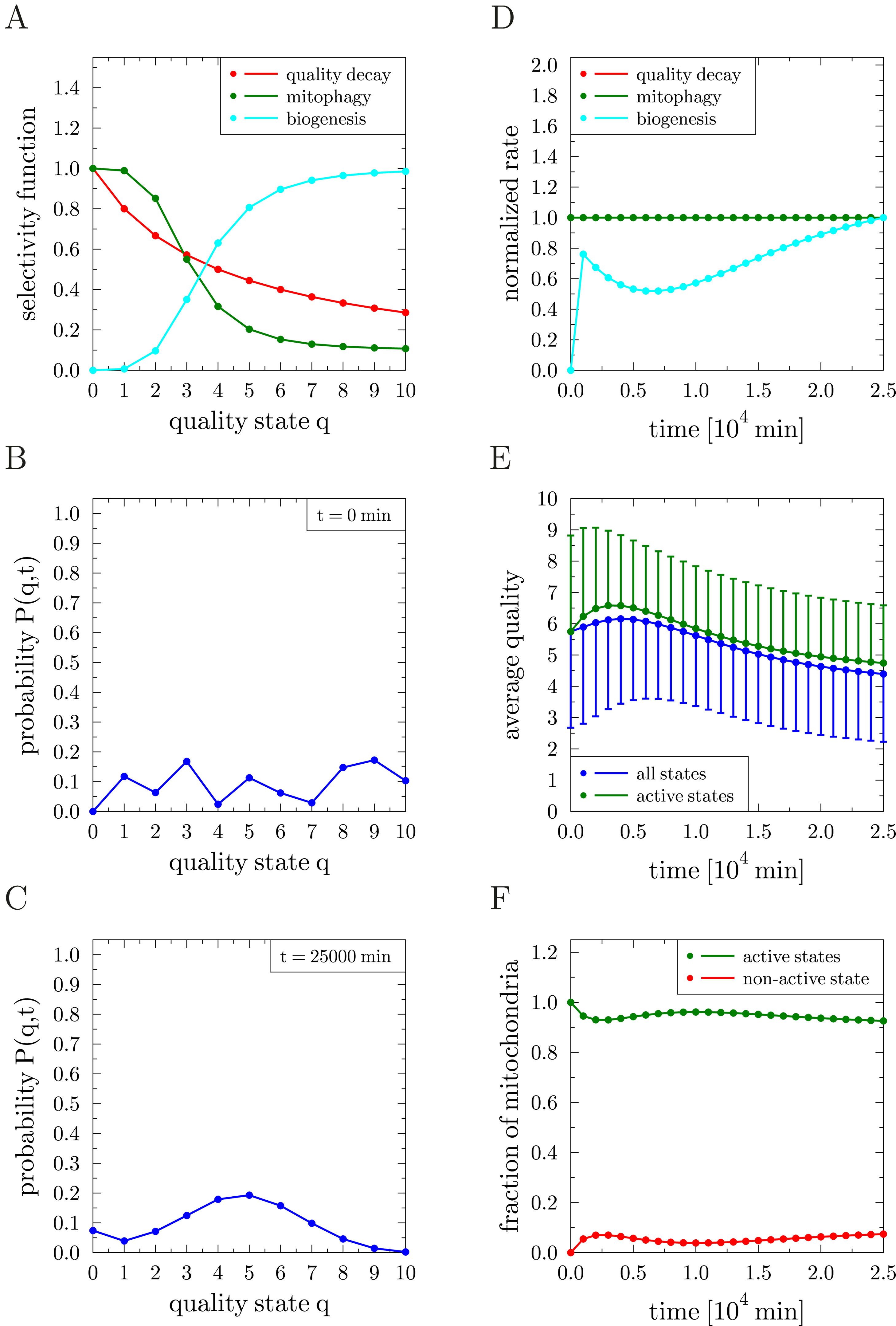

Supplement: Figure S2 — Results of the reference simulation in the absence of fusion–fission events. (A) Selectivity functions for all processes as function of quality . (B) Initial random distribution of in quality state-space at time min. (C) Probability distribution in quality state-space at time min. (D) Transition rates of all processes normalized to their individual maximal values as function of time. The red and green curves are on top of each other. (E) Average quality of mitochondria as function of time over all states (blue) and over active states (green). Error bars correspond to the standard deviation of the distribution and are plotted single-sided for reasons of clarity. (F) Fraction of mitochondria in the non-active state (red) and in active states (green) as function of time. (TIF) [file pcbi.1002576.s002.tif]

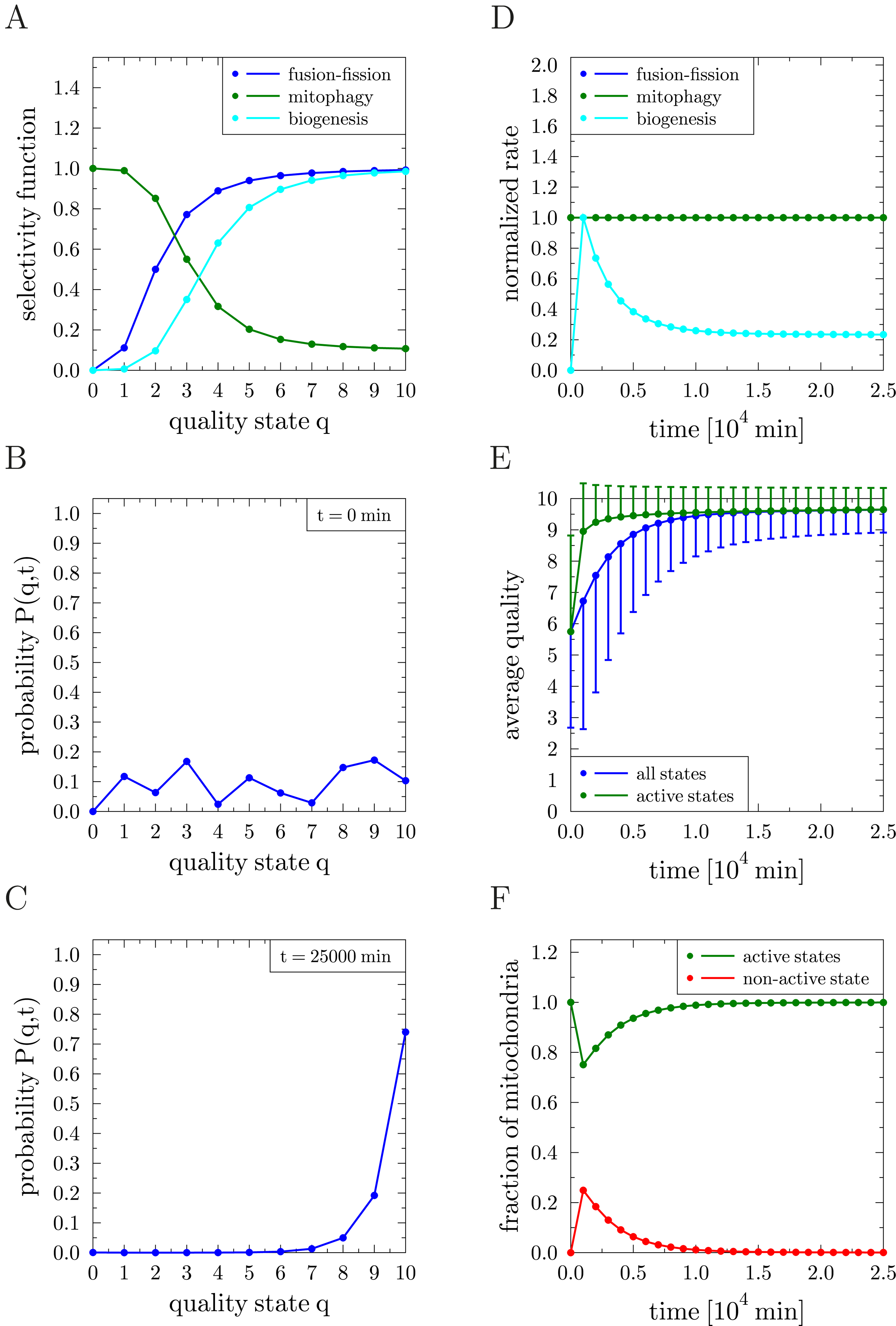

Supplement: Figure S3 — Results of the reference simulation in the absence of quality decay. (A) Selectivity functions for all processes as function of quality . (B) Initial distribution of in quality state-space at time min. (C) Equilibrium distribution of in quality state-space at time min. (D) Transition rates of all processes normalized to their individual maximal values as function of time. The blue and green curves are on top of each other. (E) Average quality of mitochondria as function of time over all states (blue) and over active states (green). Error bars correspond to the standard deviation of the distribution and are plotted single-sided for reasons of clarity. (F) Fraction of mitochondria in the non-active state (red) and in active states (green) as function of time. (TIF) [file pcbi.1002576.s003.tif]

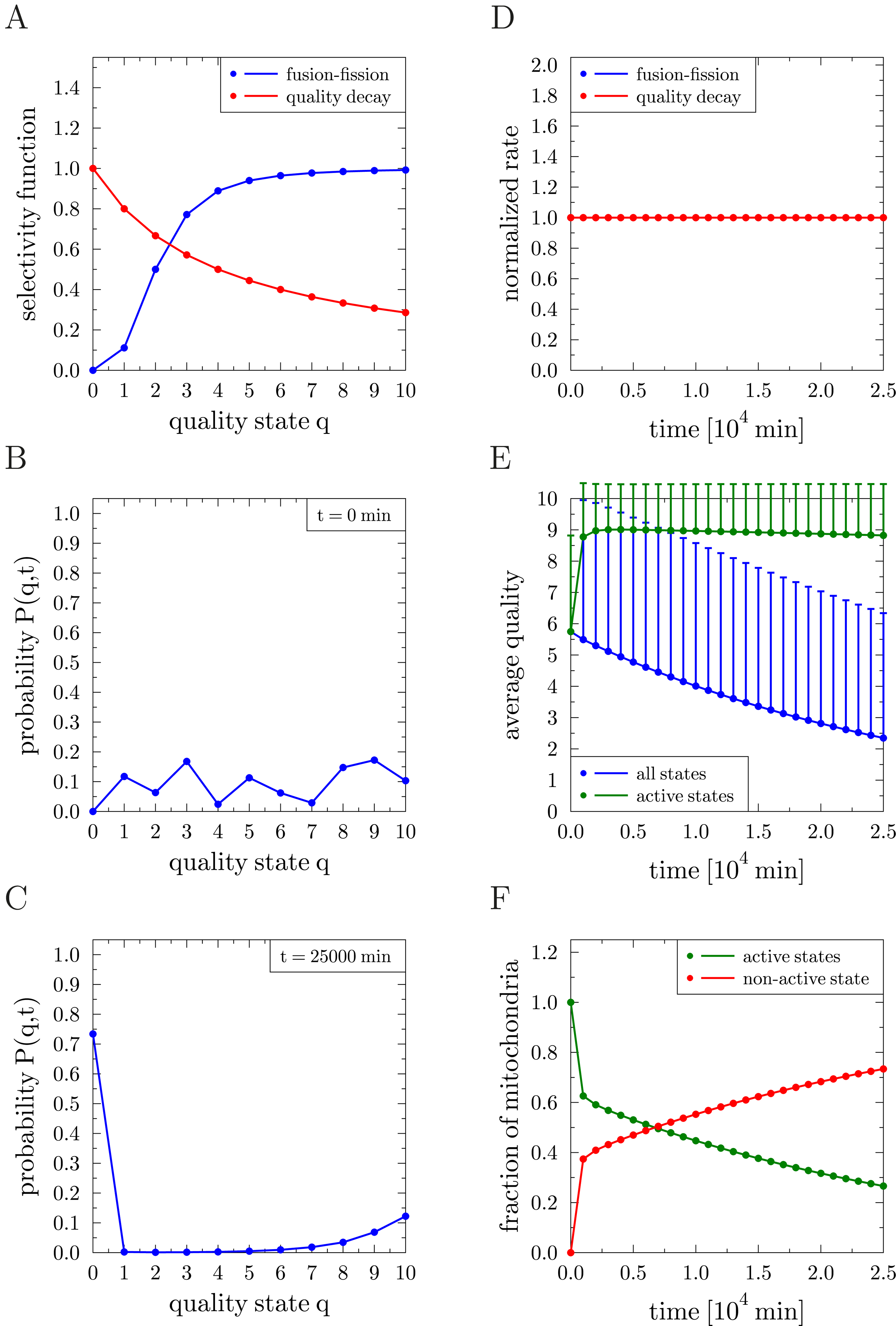

Supplement: Figure S4 — Results of the reference simulation in the absence of mitophagy and mitochondrial biogenesis. (A) Selectivity functions for all processes as function of quality . (B) Initial random distribution of in quality state-space at time min. (C) Probability distribution in quality state-space at time min. (D) Transition rates of all processes normalized to their individual maximal values as function of time. The blue and red curves are on top of each other. (E) Average quality of mitochondria as function of time over all states (blue) and over active states (green). Error bars correspond to the standard deviation of the distribution and are plotted single-sided for reasons of clarity. (F) Fraction of mitochondria in the non-active state (red) and in active states (green) as function of time. (TIF) [file pcbi.1002576.s004.tif]

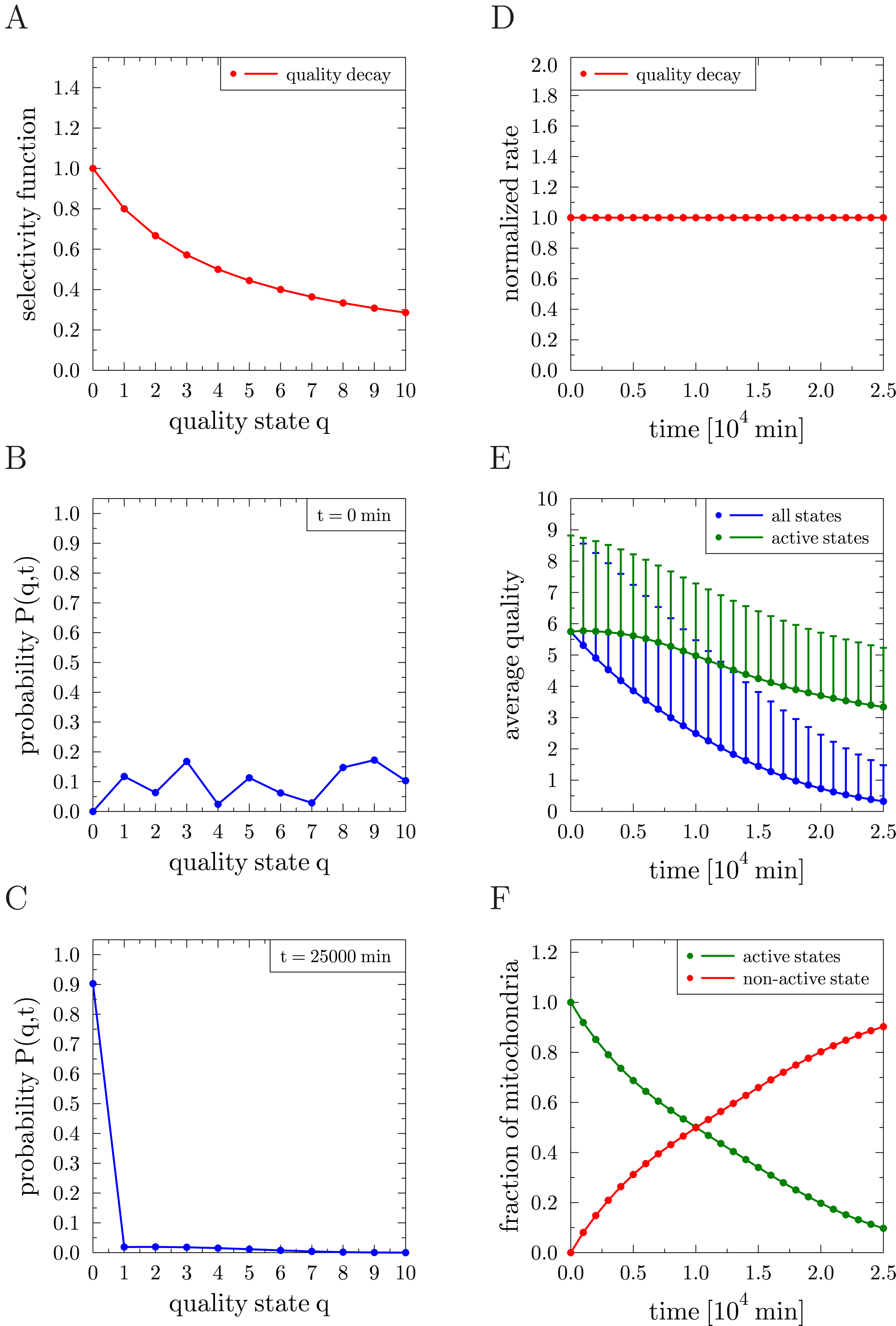

Supplement: Figure S5 — Results of the reference simulation in the absence of fusion–fission events, mitophagy and mitochondrial biogenesis. (A) Selectivity functions for all processes as function of quality . (B) Initial random distribution of in quality state-space at time min. (C) Probability distribution in quality state-space at time min. (D) Transition rates of all processes normalized to their individual maximal values as function of time. (E) Average quality of mitochondria as function of time over all states (blue) and over active states (green). Error bars correspond to the standard deviation of the distribution and are plotted single-sided for reasons of clarity. (F) Fraction of mitochondria in the non-active state (red) and in active states (green) as function of time. (TIF) [file pcbi.1002576.s005.tif]

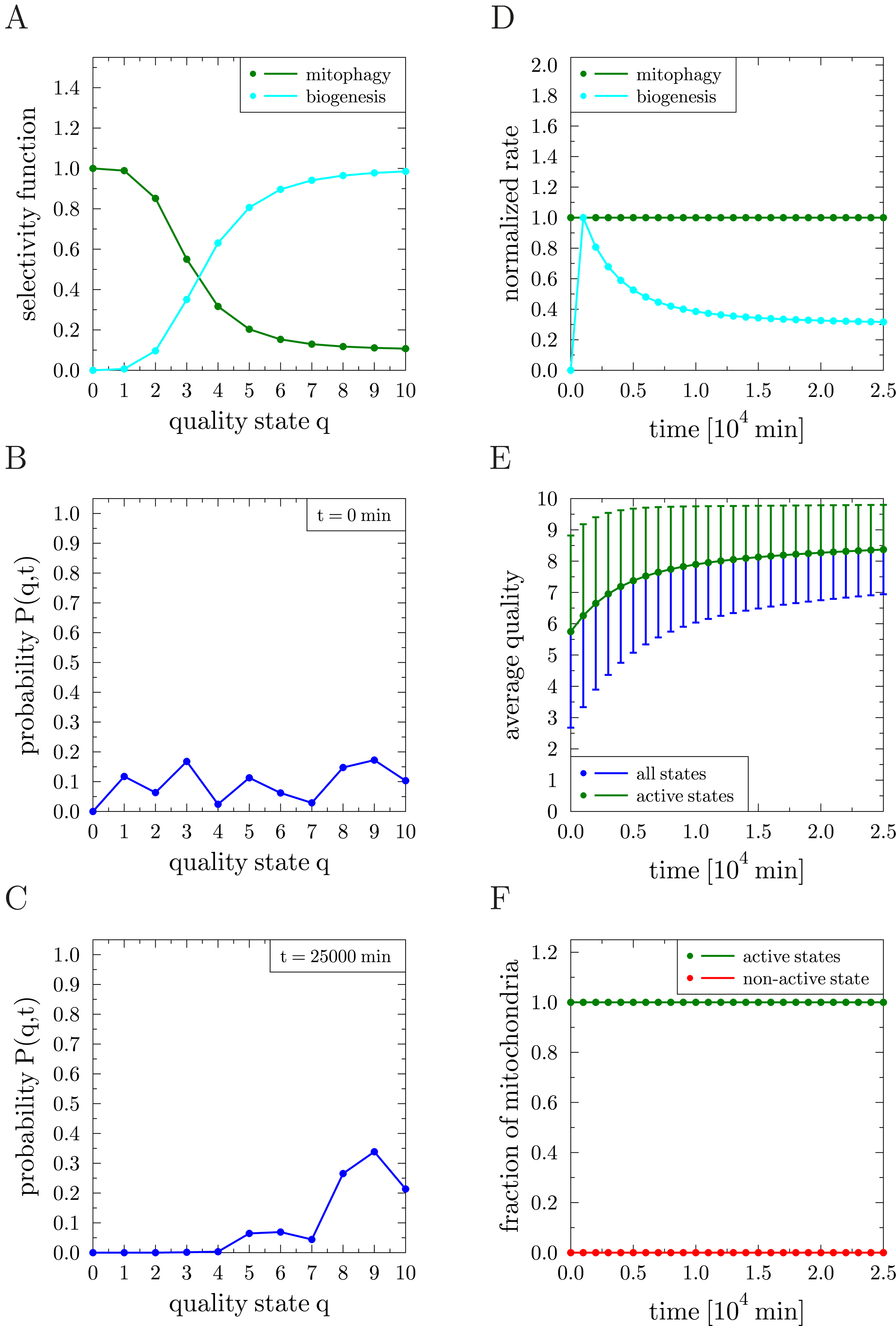

Supplement: Figure S6 — Results of the reference simulation in the absence of fusion–fission events and quality decay. (A) Selectivity functions for all processes as function of quality . (B) Initial random distribution of in quality state-space at time min. (C) Probability distribution in quality state-space at time min. (D) Transition rates of all processes normalized to their individual maximal values as function of time. (E) Average quality of mitochondria as function of time over all states (blue) and over active states (green). Error bars correspond to the standard deviation of the distribution and are plotted single-sided for reasons of clarity. (F) Fraction of mitochondria in the non-active state (red) and in active states (green) as function of time. (TIF) [file pcbi.1002576.s006.tif]

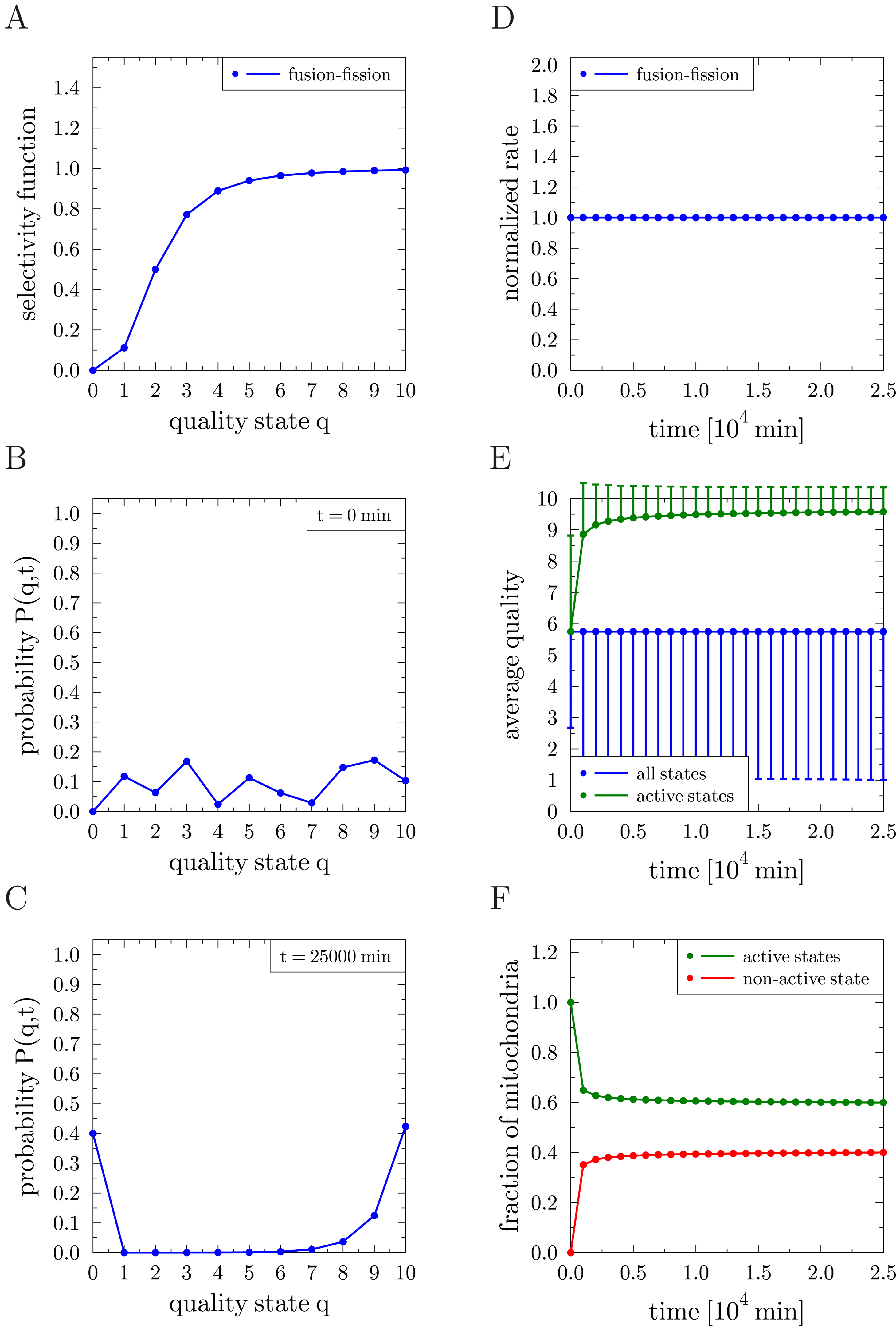

Supplement: Figure S7 — Results of the reference simulation in the absence of quality decay, mitophagy and mitochondrial biogenesis. (A) Selectivity functions for all processes as function of quality . (B) Initial random distribution of in quality state-space at time min. (C) Equilibrium distribution of in quality state-space at time min. (D) Transition rates of all processes normalized to their individual maximal values as function of time. (E) Average quality of mitochondria as function of time over all states (blue) and over active states (green). Error bars correspond to the standard deviation of the distribution and are plotted single-sided for reasons of clarity. (F) Fraction of mitochondria in the non-active state (red) and in active states (green) as function of time. (TIF) [file pcbi.1002576.s007.tif]

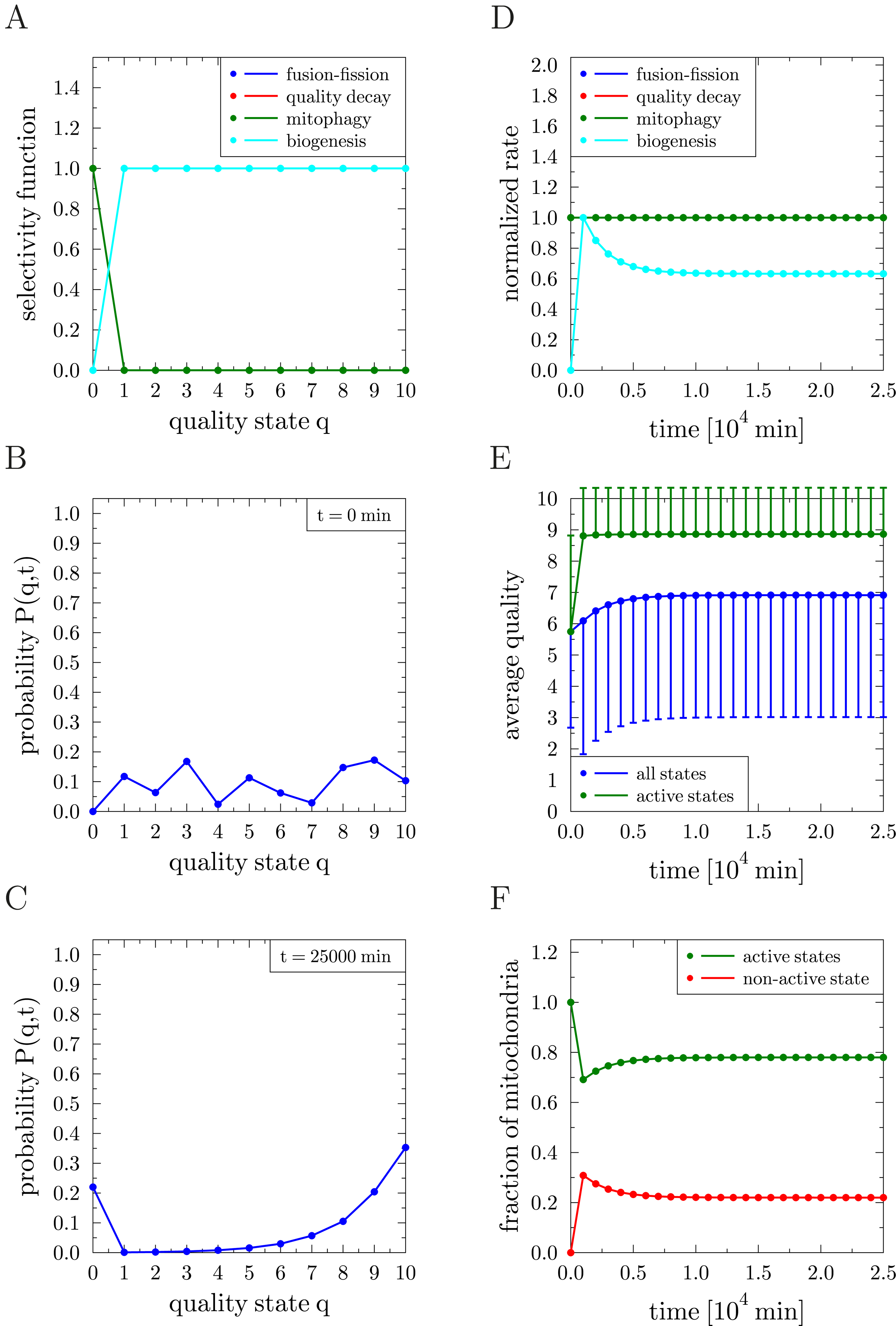

Supplement: Figure S8 — Results of the reference simulation with altered set of selectivity functions. (A) Selectivity functions for all processes as function of quality . The blue, red and cyan curves are on top of each other. (B) Initial random distribution of in quality state-space at time min. (C) Equilibrium distribution of in quality state-space at time min. (D) Transition rates of all processes normalized to their individual maximal values as function of time. The blue, red and green curves are on top of each other. (E) Average quality of mitochondria as function of time over all states (blue) and over active states (green). Error bars correspond to the standard deviation of the distribution and are plotted single-sided for reasons of clarity. (F) Fraction of mitochondria in the non-active state (red) and in active states (green) as function of time. (TIF) [file pcbi.1002576.s008.tif]

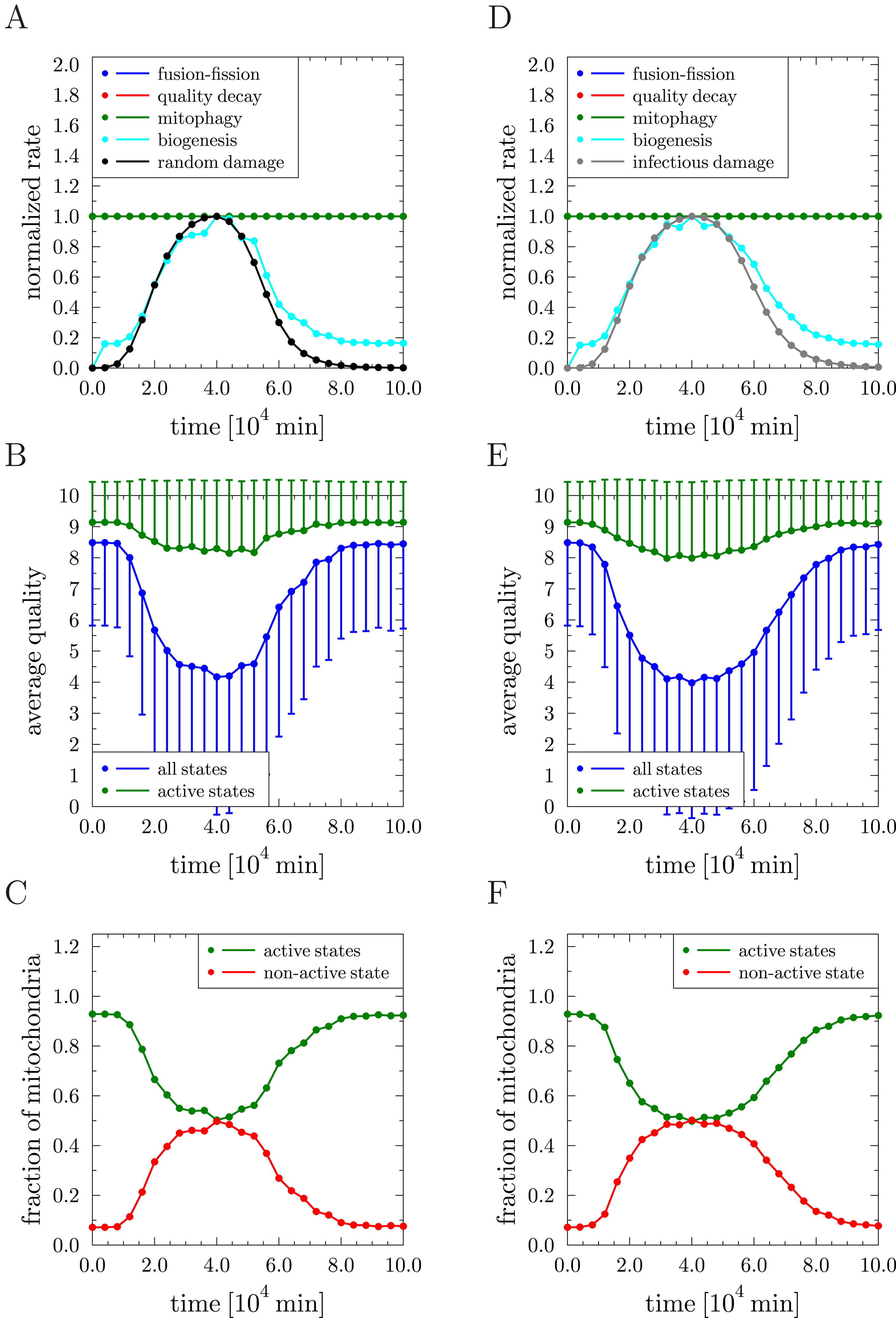

Supplement: Figure S9 — Results of the reference simulation in the presence of molecular damage as in Fig. 4 of the main text but for time-pulsed damage rates. (A)–(C) Random molecular damage: (A) Transition rates of all processes normalized to their individual maximal values as function of time. The blue, red and green curves are on top of each other. (B) Average quality of mitochondria as function of time over all states (blue) and over active states (green). Error bars correspond to the standard deviation of the distribution and are plotted single-sided for reasons of clarity. (C) Fraction of mitochondria in the non-active state (red) and in active states (green) as function of time. (D)–(F) Infectious molecular damage: the same quantities as in (A)–(C) are plotted. (TIF) [file pcbi.1002576.s009.tif]

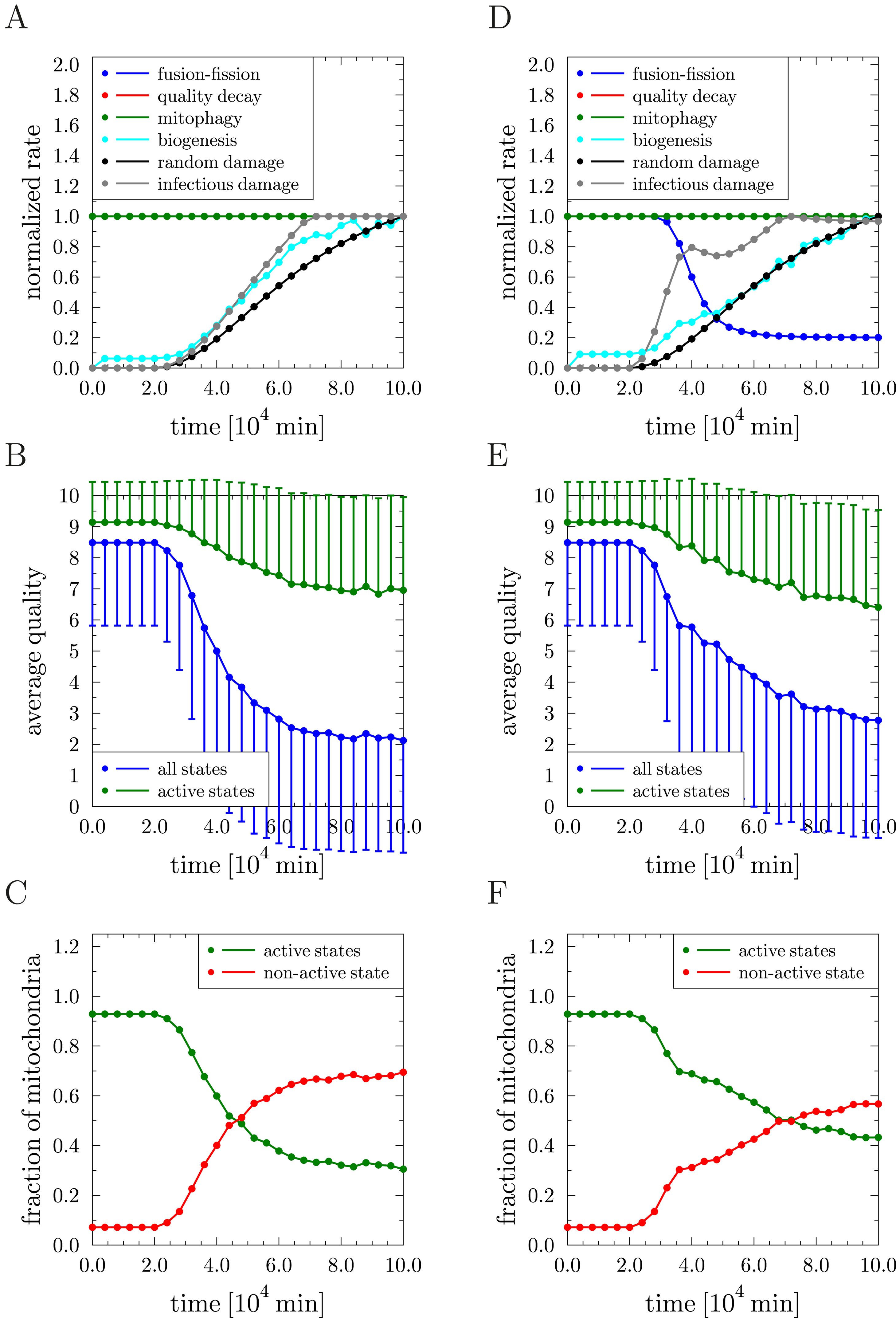

Supplement: Figure S10 — Simulation results of the MIDA model. (A)–(C) Random molecular damage triggering infectious molecular damage at a constant fusion–fission rate: (A) Transition rates of all processes normalized to their individual maximal values as function of time. The blue, red and green curves are on top of each other. (B) Average quality of mitochondria as function of time over all states (blue) and over active states (green). Error bars correspond to the standard deviation of the distribution and are plotted single-sided for reasons of clarity. (C) Fraction of mitochondria in the non-active state (red) and in active states (green) as function of time. (D)–(F) Random molecular damage triggering infectious molecular damage with time-dependent fusion–fission rate: the same quantities as in (A)–(C) are plotted. (TIF) [file pcbi.1002576.s010.tif]

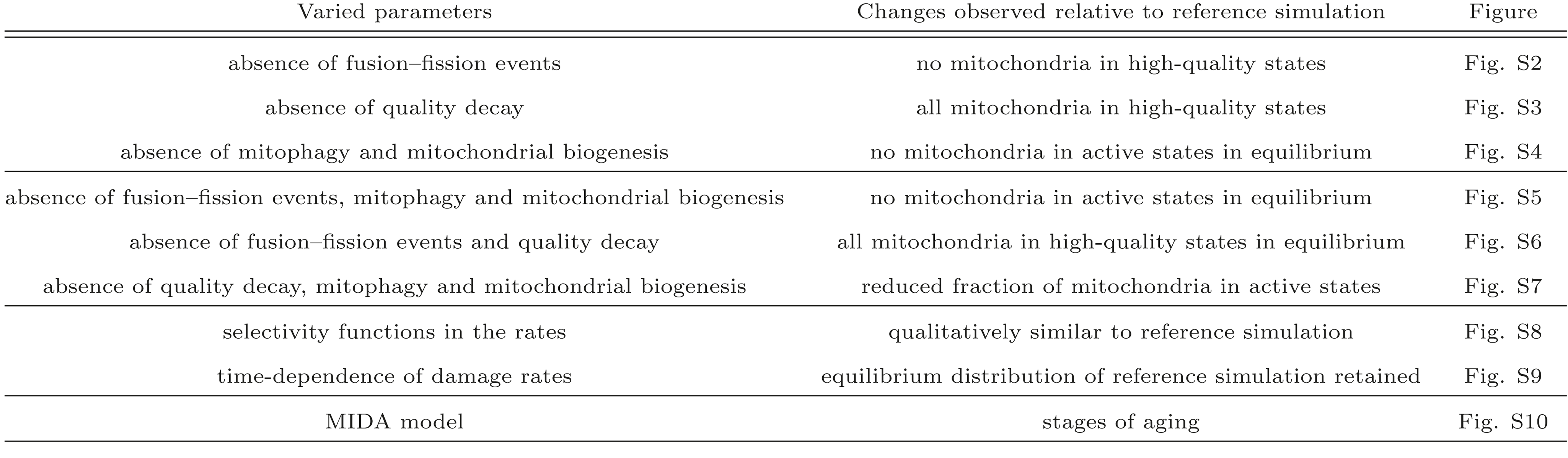

Supplement: Table S1 — Overview of results for the reference simulation with varied parameters. (TIF) [file pcbi.1002576.s011.tif]
