# Supplementary material for: Deceleration of Fusion–Fission Cycles Improves Mitochondrial Quality Control during Aging
Source: PLoS Comput Biol. 2012 Jun 28;8(6):e1002576. doi: 10.1371/journal.pcbi.1002576 (PMC3386171; doi:10.1371/journal.pcbi.1002576)
Supplement: Text S1 — Supporting information on the algorithm and additional computer simulations. (PDF) [file pcbi.1002576.s012.pdf]

Supporting Information:

Deceleration of fusion–fission cycles improves mitochondrial quality control during aging

Marc Thilo Figge[a,b,c,1], Andreas S. Reichert[d,e,1],

Michael Meyer-Hermann[c,b,2], Heinz D. Osiewacz[f,2]

[a] Applied Systems Biology, Leibniz-Institute for Natural Product Research and Infection Biology – Hans-Knöll-yInstitute and Friedrich Schiller University, 07745 Jena, Germany

[b] Frankfurt Institute for Advanced Studies, Ruth-Moufang-Str. 1, 60438 Frankfurt am Main, Germany

[c] Department of Systems Immunology, Helmholtz Centre for Infection Research, 38124 Braunschweig, Germany

[d] Mitochondrial Biology, Medical School, Goethe University Frankfurt am Main, Theodor-Stern-Kai 7, 60590 Frankfurt am Main, Germany

[e] Mitochondrial Biology, Buchmann Institute for Molecular Life Sciences, Max-von-Laue-Str. 15, 60438 Frankfurt am Main, Germany

[f] Faculty for Biosciences, Molecular Developmental Biology, Cluster of Excellence Macromolecular Complexes, Goethe University, 60590 Frankfurt am Main, Germany

[1] equally contributed

[2] corresponding authors

Algorithm

The algorithm for the time integration of the master equation was written in C++ and was based exclusively on standard C++ libraries. The source code is available from SourceForge (*http://sourceforge.net/*) under the link *http://sourceforge.net/projects/mida-model/* and the visualization of analyzed system data makes use of the Graphics Layout Engine (GLE) that is freely available under *http://glx.sourceforge.net/*.

A flow chart of the program is presented in Fig. S1. The initial system configuration is built up from the input file that contains all information about the parameter set (red box in Fig. S1). Next, to compute the system's time evolution the master equation is integrated by repeating the same procedure at every time step until the total simulation time is reached (green box in Fig. S1). Depending on the processes that are enabled in a particular simulation, e.g. absence or presence of molecular damage, the corresponding contributions to the master equation according to Eqs. (5)-(7) and (10) of the main text are taken into account in advancing the system to the next time point. The system configurations are saved in output files and are later on used to analyze the data according to Eqs. (18)-(21) of the main text and to visualize the time evolution of the system (blue box in Fig. S1.).

Additional Computer Simulations

In the sequel, we present the results of computer simulations that are obtained by varying selected parameters of the reference simulation. In Table S1, we provide an overview of the varied parameters for each simulation and a brief comment on the main impact of this variation relative to the reference simulation.

At first, we consider the impact of each process on the time-evolution of the system towards equilibrium. In order to allow for a direct comparison of the dynamics with that of the reference simulation as presented in Fig. 3 of the main text, all simulation runs were started from the same random initial distribution of mitochondria in quality state-space and were performed for the same simulation time. In cases where the equilibrium distribution was not yet reached after this simulation time, we provide additional

information on the equilibrium distribution from simulations over longer times (data not shown).

The first three simulations are performed for the reference simulation in the absence of one of the following processes: fusion-fission events (see Fig. S2), quality decay (see Fig. S3), and mitophagy and mitochondrial biogenesis (see Fig. S4).

In the absence of fusion-fission events, it is observed in Fig. S2 that mitophagy and mitochondrial biogenesis counteract quality decay. However, this does not give rise to a significant number of mitochondria in high-quality states. Even in equilibrium (data not shown) the slowly evolving system will be characterized by a significantly lower average quality of mitochondria as compared to the case where fusion-fission events are present. Thus, while mitophagy and mitochondrial biogenesis have important impact on maintaining a large number of mitochondria in active states, the molecular exchange in fusion-fission events is required to obtain mitochondria in high-quality states at reasonable time scales. This can also be seen from a comparison with Fig. S3, where we present the simulation for the reference simulation in the absence of quality decay. In this case, the system quickly evolves into an equilibrium state that is characterized by the absence of mitochondria in non-active states and occupation of high-quality states. The importance of mitophagy and mitochondrial biogenesis for maintaining the fraction of active mitochondria becomes apparent in the absence of these processes. As can be inferred from Fig. S4, in this case the system slowly evolves into an equilibrium state where no active states will be occupied (data not shown). Thus, fusion-fission events alone are not sufficient to maintain a high fraction of active mitochondria, but are required to quickly establish and maintain mitochondria in high-quality states. This is realized by fusion-fission events at the cost of the quantity of mitochondria in active states, since the induction of high-quality mitochondria is directly associated with the induction of low-quality mitochondria that tend to become non-active. Therefore, mitophagy and mitochondrial biogenesis, albeit being relatively slow processes, provide an important backbone of maintaining sufficient mitochondria in high-quality states by the much faster fusion-fission events.

Next, we perform three simulations of the reference simulation including only the process of quality

decay (see Fig. S5), mitophagy and mitochondrial biogenesis (see Fig. S6), and fusion-fission events (see Fig. S7).

We observe from Fig. S5 that quality decay alone gives rise to the expected system behavior with mitochondria accumulating in the state with $q = 0$. Since no process is counteracting the quality decay, in equilibrium there will be no mitochondria left in states with $q > 0$ (data not shown). The situation is different in the case where only mitophagy and mitochondrial biogenesis are present, as is shown in Fig. S6. In this case, quality improvement is observed, however, as the driving processes are significantly slower than fusion-fission events, an equilibrium state is only reached after simulation times that are orders of magnitude longer than presented here. In addition, it should be kept in mind that processes like quality decay (see Fig. S1) and molecular damage have a strong impact on the distribution of mitochondria in quality state-space by lowering the average quality. On the other hand, fusion-fission events ensure the fast equilibration of the system into a state with a large number of mitochondria in high-quality states, as can be inferred from Fig. S6. Again, in the absence of mitophagy and mitochondrial biogenesis this distribution is established at the cost of a larger number of mitochondria accumulating in the non-active state. This suggests that the interplay between fusion-fission events on the one hand and mitophagy and mitochondrial biogenesis on the other hand is important for the establishment and maintenance of a mitochondrial distribution that this characterized by both, large quantity and high quality of active mitochondria (see Fig. S3).

To demonstrate the robustness of the qualitative results that we obtained from the reference simulation, we show in Fig. S8 the simulation results for a varied set of selectivity functions, i.e. we changed the rates regarding their quality-dependence from smooth Hill functions into abruptly changing functions of q. The selectivity functions for fusion-fission events, quality decay and mitochondrial biogenesis were chosen to have the same value for all quality states of active mitochondria ($q > 0$). Moreover, these three processes do not occur for mitochondria in the non-active quality state ($q = 0$), whereas mitophagy only occurs for mitochondria in the non-active state. The altered selectivity functions are plotted in

Fig. S8A and all other parameters are the same as in the reference simulation. Starting from the same random initial distribution (see Fig. S8B), we obtain the equilibrium distribution of mitochondria in quality state-space that is shown in Fig. S8C. The average quality of mitochondria (see Fig. S8E) and the fractions of mitochondria in active and non-active states (see Fig. S8F) are found to be qualitatively similar to that in the reference simulation, despite the rather drastic change in the selectivity functions. Quantitative differences are observed, *e.g.* with regard to the time scale on which the flow equilibrium is reached. In the present case, the system reaches the equilibrium state faster and the dynamically adapting renewal rate (see Fig. S8D) attains the equilibrium value $r_r(t \to \infty) = 1.1 \times 10^{-4}$ min^{-1}, which is slightly higher than in the reference simulation ($r_r(t \to \infty) = 8.1 \times 10^{-5}$ min^{-1}). It can be concluded from this and other tested variations of the selectivity functions (data not shown) that the precise profile of these Hill functions does not change the qualitative conclusions drawn from the reference simulation with its particular choice of parameters for the sensitivity functions.

Similarly, we checked the robustness of the reference simulation with regard to the time-dependence of the rates. We generally observe that the system attains the same equilibrium configuration for the same set of parameters, independent of the profile of the Hill function that defines the continuous transition between two states. By way of exmaple, this is illustrated in Fig. S9, where we repeat the simulations of Fig. 4 in the main text for the reference system in the presence of molecular damage. However, in contrast to the simulations in Fig. 4, the time-dependence of the rate for molecular damage is not simply given by a monotonically increasing Hill function but by a combination of two Hill functions that give rise to a pulse in the rate of molecular damage. This can be seen in Fig. S9A-C and Fig. S9D-E, respectively, for random molecular damage and for infectious molecular damage. The simulation is started from the equilibrium state of the reference simulation and during the first part of the simulations, *i.e.* until the maximum in the damage rates is reached, the dynamic change of the system is in agreement with the time-evolution of the simulations in Fig. 4, as expected. Then, when the damage rates are declining and reaching again zero values, the system is observed to evolve back into the inital state, *i.e.* the

equlibrium state of the reference simulation. The parameters that determine the time-pulsed damage rates are generally observed to determine the system kinetics, *e.g.* regarding the time scale on which the system equilibrates, rather than altering the equilibrium state.

Finally, we perform a simulation of the MIDA model that was started from the equilibrated reference simulation in order to demonstrate that deceleration of fusion–fission cycles improves mitochondrial quality control during aging. In Fig. S10 we show simulation results obtained by either keeping the fusion–fission rate constant in time (Fig. S9A-C) or allowing its dynamic decrease in time (Fig. S9D-F). In both cases, the dynamical increase of random molecular damage is starting at $t = 2 \cdot 10^4$min, where the parameters of the Hill function are chosen to be $r_{rd}(0) = 0\,\text{min}^{-1}$, $r_{rd}(\infty) = 7.5 \cdot 10^{-2}\text{min}^{-1}$, $\tau_{rd} = 5 \cdot 10^4$min, $h_{rd} = 2$, and the random fraction is set to $f_{rd} = 3\%$ (see Eqs. (11) and (12)). The infectious molecular damage is induced according to Eq. (25) with $\bar{r}_{rd} = 0.05\ \text{min}^{-1}$ and at $t = 2.5 \cdot 10^4$min we allow the fusion–fission rate to adapt according to a Hill function with parameters $r_{ff}(0) = 5 \cdot 10^{-2}\,\text{min}^{-1}$, $r_{ff}(\infty) = 1 \cdot 10^{-2}\text{min}^{-1}$, $\tau_{ff} = 1 \cdot 10^4$min, and $h_{ff} = 4$. All other parameters are chosen as in the reference simulation and the simulation is started from the equilibrium distribution of $P(q,t)$ as obtained from the reference simulation.

The following three stages may be distinguished:

Stage I: During this stage molecular damage is absent implying and the system dynamics is equivalent in Fig. S10A-C and Fig. S10D-F.

Stage II: At time $t = 2 \cdot 10^4$min, random molecular damage occurs that induces infectious molecular damage. This gives rise to a fast decrease in the average quality (see Fig. S10B and Fig. S10E) and in the fraction of active mitochondria (see Fig. S10C and Fig. S10F). However, allowing for the dynamic deceleration of fusion–fission events starting at time $t = 2.5 \cdot 10^4$min, gives rise to an improvement in the mitochondrial quality control. Higher values for the average quality and the fraction of active mitochondria are maintained in this case (see Fig. S10E and Fig. S10F) as compared to the case of constant fusion–fission rate (see Fig. S10B and Fig. S10C).

Stage III: Since the funtioning of a cell requires the fraction of active mitochondria to be maintained above a survival threshold, arbitrarily set to the point where the fraction of active and non-active mitochondria become equal. For the simulation with constant fusion–fission rate this survival threshold is reached at time $t = 4.5 \cdot 10^4$min in Fig. S10C, however, in the MIDA model cellular survival is prolonged until $t = 7 \cdot 10^4$min in Fig. S10F.

We conclude that the duration of Stage II is increased by a factor two in the case of the MIDA model as compared to the non-MIDA model with constant fusion–fission rate.

Tables

Varied parameters	Changes observed relative to reference simulation	Figure
absence of fusion–fission events	no mitochondria in high-quality states	Fig. S2
absence of quality decay	all mitochondria in high-quality states	Fig. S3
absence of mitophagy and mitochondrial biogenesis	no mitochondria in active states in equilibrium	Fig. S4
absence of fusion–fission events, mitophagy and mitochondrial biogenesis	no mitochondria in active states in equilibrium	Fig. S5
absence of fusion–fission events and quality decay	all mitochondria in high-quality states in equilibrium	Fig. S6
absence of quality decay, mitophagy and mitochondrial biogenesis	reduced fraction of mitochondria in active states	Fig. S7
selectivity functions in the rates	qualitatively similar to reference simulation	Fig. S8
time-dependence of damage rates	equilibrium distribution of reference simulation retained	Fig. S9
MIDA model	stages of aging	Fig. S10

Table S1: Overview of results for the reference simulation with varied parameters.

Table S1.
